# An extracellular vesicle epitope profile is associated with acute myocardial infarction

**DOI:** 10.1111/jcmm.15594

**Published:** 2020-07-14

**Authors:** Jacopo Burrello, Sara Bolis, Carolina Balbi, Alessio Burrello, Elena Provasi, Elena Caporali, Lorenzo Grazioli Gauthier, Andrea Peirone, Fabrizio D'Ascenzo, Silvia Monticone, Lucio Barile, Giuseppe Vassalli

**Affiliations:** ^1^ Laboratory of Cellular and Molecular Cardiology Cardiocentro Ticino and Foundation for Cardiovascular Research and Education (FCRE) Lugano Switzerland; ^2^ Laboratory for Cardiovascular Theranostics Cardiocentro Ticino and Foundation for Cardiovascular Research and Education (FCRE) Lugano Switzerland; ^3^ Department of Electrical Electronic and Information Engineering "Guglielmo Marconi" (DEI) University of Bologna Bologna Italy; ^4^ Division of Cardiology Department of Medical Sciences University of Torino Torino Italy; ^5^ Division of Internal Medicine Department of Medical Sciences University of Torino Torino Italy; ^6^ Faculty of Biomedical Sciences Università della Svizzera Italiana (USI) Lugano Switzerland; ^7^ Institute of Life Science Scuola Superiore Sant'Anna Pisa Italy; ^8^ Center for Molecular Cardiology University of Zurich Zurich Switzerland

**Keywords:** acute myocardial infarction, biomarker, coronary artery disease, extracellular vesicles, machine learning, ST‐segment elevation myocardial infarction

## Abstract

The current standard biomarker for myocardial infarction (MI) is high‐sensitive troponin. Although powerful in clinical setting, search for new markers is warranted as early diagnosis of MI is associated with improved outcomes. Extracellular vesicles (EVs) attracted considerable interest as new blood biomarkers. A training cohort used for diagnostic modelling included 30 patients with STEMI, 38 with stable angina (SA) and 30 matched‐controls. Extracellular vesicle concentration was assessed by nanoparticle tracking analysis. Extracellular vesicle surface‐epitopes were measured by flow cytometry. Diagnostic models were developed using machine learning algorithms and validated on an independent cohort of 80 patients. Serum EV concentration from STEMI patients was increased as compared to controls and SA. EV levels of CD62P, CD42a, CD41b, CD31 and CD40 increased in STEMI, and to a lesser extent in SA patients. An aggregate marker including EV concentration and CD62P/CD42a levels achieved non‐inferiority to troponin, discriminating STEMI from controls (AUC = 0.969). A random forest model based on EV biomarkers discriminated the two groups with 100% accuracy. EV markers and RF model confirmed high diagnostic performance at validation. In conclusion, patients with acute MI or SA exhibit characteristic EV biomarker profiles. EV biomarkers hold great potential as early markers for the management of patients with MI.

## 1 INTRODUCTION

Coronary artery disease is the most frequent cause of death worldwide.^1^ Among acute coronary syndromes, ST‐segment elevation myocardial infarction (STEMI) is associated with a 12%‐mortality within 6 months.^2^ Early diagnosis of myocardial infarction (MI) is critical to preserve cardiac function and improve outcomes.^3^, ^4^ This diagnosis currently relies on chest pain suggestive of myocardial ischemia, specific electrocardiogram (ECG) changes, and detection of increased serum levels of high‐sensitive (hs) troponin,^4^ which is released within 2‐3 hours after the onset of cardiac injury. Although extremely useful in a daily clinical setting, a search for novel biomarkers to identify patients with MI early after onset of symptoms, when primary percutaneous coronary intervention (PCI) has the greatest chance of influencing their outcomes, is warranted.^5^, ^6^

Emerging potential biomarkers for coronary artery disease (CAD) include secreted extracellular vesicles (EVs), which are found in all biological fluids including peripheral blood.^6^, ^7^, ^8^ Their diagnostic power derives from the enrichment of potential protein markers which otherwise constitute only a very small proportion (<0.01%) of the total proteome of body fluids.^9^ EVs can be subdivided into exosomes, which originate from the endosomal system, and microvesicles, which directly shed by plasma membranes. Traditionally, EVs were subdivided by size: small EVs (30 nm‐150 nm in diameter) mainly consisting of exosomal EVs, and medium‐large EVs (150 nm‐1000 nm in diameter) mainly representing microvesicles. Some EV cargo proteins specify a cellular origin or change in amount in certain diseases, so that they can serve as biomarkers of diseases. Exosomal surface epitopes include the tetraspanins CD9, CD63, and CD81. Intra‐vesicular exosomal markers include tumour susceptibility gene 101 (TSG101).^6^, ^10^

Most circulating EVs in healthy individuals are derived from platelets and erythrocytes; however, vesicles also are released from leucocytes, endothelial cells, monocytes, neutrophils and lymphocytes.^7^, ^11^ Previous studies have shown increased numbers of platelet, endothelial cell‐ and leucocyte‐derived EVs in patients with acute coronary syndromes (ACS) or chronic CAD.^12^, ^13^, ^14^, ^15^, ^16^, ^17^, ^18^, ^19^, ^20^, ^21^, ^22^, ^23^ However, partially divergent results have been reported, in part due to methodological differences in EV isolation.^24^, ^25^ Moreover, previous analyses focused on a few surface epitopes of interest, rather than on the EV epitope surface profile.

The main potential advantage of EV profiling in the management of patients with CAD is represented by the very early increase of circulating particles released by suffering but still alive cardiomyocytes, during heart ischemia.^6^, ^22^ Indeed, the increase in EV‐derived biomarkers may even anticipate the raise of hs‐troponin, which need cardiomyocytes death. However, EV profiling needs for protocol standardization and technology implementation, and that this approach remains to date relatively time‐consuming and expensive when compared to troponin.^6^ Potential advantages and disadvantages of EV profiling as compared to hs‐troponin in the management of patients with CAD are summarized in Table S1.

In the present study, we analysed a comprehensive panel of 37 EV surface epitopes, representative of a variety of cells of origin, in serum samples from patients with acute MI and patients with chronic CAD presenting with stable angina (SA), compared to age‐ and sex‐matched healthy controls, using a validated multiplex flow cytometry (FC) assay.^25^, ^27^ A random forest (RF) model based on EV surface epitopes accurately discriminated STEMI patients and controls.

## 2 METHODS

A detailed description of patient data, EV extraction and characterization protocols, statistical analyses and diagnostic modelling is provided in the Appendix S1.

### 2.1 Participants and blood sampling

We analysed peripheral venous blood samples collected from individuals recruited at the Fondazione Cardiocentro Ticino, Lugano (Switzerland), as a training cohort for diagnostic modelling purposes. An independent cohort enrolled at Città della Salute e della Scienza, University of Torino (Italy), served as validation cohort. The study protocol was approved by the local ethical committees. All participants gave informed written consent to the study in accordance with the declaration of Helsinki. Peripheral venous blood samples were collected from patients presenting with a diagnosis of STEMI, according to the European Society of Cardiology (ESC) guidelines,^4^ on presentation to the emergency department before primary PCI, as well as 24 hours (hours) and 48 hours thereafter. Exclusion criteria included: (a) Chest pain onset ≥6 hours; (b) Age > 85; (c) Cardiac arrest or cardiogenic shock with indication to invasive device assistance; (d) Glomerular filtration rate <30 mL/min; (e) Atrial fibrillation, ventricular tachycardia, ventricular fibrillation or other arrhythmias requiring defibrillation; (f) Non‐ischemic heart disease such as severe heart valve disease, chronic heart failure and other heart diseases with impaired left ventricular ejection fraction (LVEF); (g) Acute or chronic inflammatory diseases (eg auto‐immune disease, cancer and infections). In addition, samples were collected from patients with chronic CAD presenting with SA according to ESC guidelines,^28^ with angiographically documented coronary stenoses (≥70% internal vessel diameter reduction). Asymptomatic subjects who underwent 64‐slice multidetector computed tomography of coronary arteries for primary prevention purposes and had documented absence of coronary stenoses (ie >30% diameter reduction), were enrolled as controls. The aforementioned exclusion criteria also applied to SA patients and controls. Unblinded investigators were responsible for identifying participants but were not involved in any experimental procedures.

### 2.2 Sample processing

Blood was collected in 7 mL heparin‐ and EDTA‐free polypropylene tubes. The first blood tube was discarded. Blood was centrifuged at 1600 g for 15 minutes at 4°C, and supernatant was centrifuged at 3000 g for 20 minutes, 10 000 g for 15 minutes and 20 000 g for 30 minutes to remove intact cells, cellular debris and larger EVs (apoptotic bodies and EV aggregates; see also Extended Methods and Figure S1A). After centrifugation steps, supernatant was divided into 0.1 mL aliquots and stored at −80°C until analysis. Pre‐analytical factors for blood collection and storage complied with guideline for EV biomarkers.^6^

### 2.3 Western blot analysis

Western blot analysis was performed on 100 μL of serum samples incubated overnight with MACSPlex capture beads. Next day, the unbounded fraction was discarded, and samples were lysed with RIPA Buffer. Total proteins (15 μg) were separated on SDS Page 4%‐12% gel (BioRad) and transferred onto PVDF membrane. The blot was incubated with the following primary antibody: Rabbit monoclonal anti‐TSG101 (ab125011 Abcam); mouse monoclonal anti‐CD81 (MA5‐17937 Invitrogen); rabbit polyclonal anti‐apolipoprotein A1 (ab33470 Abcam) and rabbit polyclonal anti‐apolipoprotein B48 (ab31992 Abcam).

### 2.4 Nanoparticle tracking analysis (NTA)

To measure serum particle concentration and diameter, we used NTA with NanoSight LM10 (Malvern Instruments) equipped with a 405 nm laser and NTA 2.3 analytic software. EV concentration is shown as particle number/mL (median value and interquartile range).

### 2.5 EV surface epitope analysis

Serum samples underwent bead‐based EV immunocapture and were analysed by FC using MACSPlex human Exosome Kit (Miltenyi; Bergisch Gladbach, Germany), as detailed in the Appendix S1 (see also Figure S1A). Briefly, serum supernatant was incubated with 37 fluorescently labelled capture bead populations (Table S2), each coated with a specific antibody binding the respective surface epitope, and 2 control bead populations, followed by the EV detection reagent (ie fluorescently labelled antibodies for CD9/CD63/CD81). Median fluorescence intensity (MFI) was measured on a MACSQuant‐Analyzer‐10 flow cytometer (Miltenyi) according to previous validation studies.^26^, ^27^ All markers were analysed simultaneously. Surface epitope levels were referenced to EV‐specific epitopes by subtracting the respective fluorescence values of blank control from MFI values for individual surface epitopes, and by normalizing them for CD9/CD63/CD81 MFI, reflecting EV concentration. To rule out confounding effects of the protocol used, this method was compared with alternate protocols in a small subset of patients. Specifically, the effect of EV isolation by ultracentrifugation or size‐exclusion chromatography (SEC) was assessed (Figure S2A,B). Moreover, EV markers were determined in both serum and plasma samples from the same patients (Figure S2C and S3). A shortened incubation time (1 hour) of serum supernatant with capture beads was tested as compared to overnight incubation (Figure S2D). Finally, technical reproducibility of the assay was confirmed by analysing twice the same sample (Figure S4).

### 2.6 Statistical analysis and diagnostic modelling

IBM SPSS Statistics 22 (IBM), Python 3.5 (library, scikit‐learn) and GraphPad PRISM 7.0a were used for statistical analyses. ANOVA with post hoc Bonferroni's test and Kruskal‐Wallis's test was used to compare variables with a normal or non‐normal distribution, respectively. Categorical variables were compared through a chi‐square test. Correlations were evaluated by Pearson's test and regression curve analyses. The analysis of receiver operating characteristics (ROC) curves was used to compare diagnostic performances of selected variables. Multivariate logistic regression analysis was performed to determine odds ratio (ORs). *P*‐values < .05 were considered significant.

Supervised machine learning algorithms and in particular linear discriminant analysis (LDA) and random forest (RF) models have been exploited in the context of diagnostic modelling for clinical research, as described previously.^29^, ^30^ LDA was used as a strategy for feature reduction to build the canonical plot. Diagnostic models were built through a RF classification algorithm; the algorithm created 20 different classification trees and the predicted diagnosis resulted from the outcome of each tree of the forest. Diagnostic performance and generalizability of the models developed in the training cohort were validated in an independent cohort.

## 3 RESULTS

### 3.1 Clinical and biochemical characteristics of the study cohorts

We analysed 238 serum samples collected from 178 participants from a training cohort (n = 98) and an independent validation cohort (n = 80). The training cohort was divided into 3 groups (STEMI: n = 30, mean [SD] age, 63 [12.5] years; 6 women [20.0%]; SA: n = 38, mean [SD] age, 65 [8.9] years; 9 women [23.7%]; controls: n = 30, mean [SD] age, 60 [9.8] years; 12 women [40.0%]; Table 1). Dyslipidemia (48.0%) and hypertension (43.9%) were highly prevalent in the study population, followed by smoking (14.3%), diabetes (12.2%) and chronic kidney disease (5.1%). Study groups did not significantly differ from one another with respect to age, sex, body weight, body‐mass index, prevalence of cardiovascular risk factors, systolic and diastolic blood pressure, renal function, lipid profile and glucose levels. STEMI patients significantly differed from SA patients and controls only with respect to hs‐troponin, white blood cells (WBC), C‐reactive protein (CRP) and left ventricular ejection fraction (LVEF) assessed by echocardiography <24 hours post‐PCI. Infarcted myocardial regions and culprit vessels are described in Table S3. The time interval (median [range]) between onset of chest pain and blood sampling in STEMI patients was 2.75 [2.0‐4.0] hours. Pharmacological treatment at the enrolment was not significantly different between groups (Table S4). Based on the results in the training cohort, EV markers were validated for the diagnosis of STEMI, in an independent cohort. Clinical and biochemical characteristics of patients in the validation cohort did not significantly differ from the training cohort (Table S5). Except for cardiac‐specific hs‐troponin I, WBC counts and LVEF, patients' characteristics were not significantly different in STEMI patients and controls in the validation cohort (Table S6).

**Table 1:** Clinical and biochemical characteristics (training cohort)

Variable	CTRL [n = 30]	STEMI [n = 30]	SA [n = 38]	Overall *P‐*value	Pairwise comparisons		
					CTRL vs STEMI	CTRL vs SA	STEMI vs SA
Age (y)	60 ± 9.8	63 ± 12.5	65 ± 8.9	.219	–	–	–
Sex (ref. male)	18 (60.0)	24 (80.0)	29 (76.3)	.176	–	–	–
Familiarity for CAD (%)	11 (36.7)	6 (20.0)	15 (39.5)	.201	–	–	–
Hypertension (%)	9 (30.0)	13 (43.3)	21 (55.3)	.114	–	–	–
Diabetes (%)	1 (3.3)	4 (13.3)	7 (18.4)	.165	–	–	–
Dyslipidemia (%)	12 (40.0)	14 (46.7)	21 (55.3)	.451	–	–	–
CKD (%)	2 (6.7)	2 (6.7)	1 (2.6)	.676	–	–	–
Smoking Habit (%)	2 (6.7)	4 (13.3)	8 (21.1)	.370	–	–	–
Systolic BP (mm Hg)	130 ± 14.1	134 ± 23.4	136 ± 17.2	.523	–	–	–
Diastolic BP (mm Hg)	81 ± 5.0	82 ± 14.7	79 ± 8.2	.714	–	–	–
Weight (kg)	75 ± 9.1	77 ± 14.9	79 ± 17.7	.619	–	–	–
BMI (kg/m^2^)	26.4 ± 3.27	26.3 ± 4.25	27.3 ± 5.4	.631	–	–	–
hs‐troponin (ng/L)	8 ± 11.1	669 ± 1295.0	10 ± 14.6	**.002**	**.012**	1.000	**.004**
WBC (n/L)	7108 ± 2220.0	10 583 ± 2535.3	7439 ± 2244.9	**<.001**	**<.001**	1.000	**<.001**
Creatinine (mg/dL)	0.89 ± 0.236	0.95 ± 0.196	0.94 ± 0.152	.543	–	–	–
GFR (mL/min)	90 ± 14.3	88 ± 31.9	81 ± 29.8	.490	–	–	–
CRP (mg/L)	1.8 ± 0.78	7.6 ± 7.30	5.0 ± 5.38	**.002**	**.001**	.119	.161
Glycemia (mmol/L)	6.6 ± 2.56	8.3 ± 2.98	7.2 ± 2.59	.082	–	–	–
Total cholesterol (mmol/L)	4.8 ± 0.73	5.0 ± 1.47	4.5 ± 1.03	.283	–	–	–
HDL (mmol/L)	1.4 ± 0.42	1.6 ± 1.66	1.3 ± 0.46	.595	–	–	–
Triglycerides (mmol/L)	1.5 ± 1.18	1.5 ± 1.98	1.3 ± 0.65	.805	–	–	–
LVEF at echo (%)	62 ± 4.3	52 ± 8.4	60 ± 7.0	**<.001**	**<.001**	.614	**<.001**

Note

Clinical and biochemical characteristics of patients diagnosed with ST‐segment elevation myocardial infarction (STEMI; n = 30) compared to controls (CTRL; n = 30) and patients with stable angina (SA; n = 38), who were enrolled in the training cohort. Data are expressed as mean ± SD, or absolute number (percentage), when appropriated. *P‐*values < .05 were considered significant and indicated by bold characters.

Abbreviations: BP, Blood Pressure; CAD, Coronary Artery Disease; CKD, Chronic Kidney Disease; CRP, C‐Reactive Protein; GFR, Glomerular Filtration Rate; LVEF, Left Ventricular Ejection Fraction at echocardiography; WBC, White Blood Cells.

### 3.2 EV number and size

**FIGURE 1:**
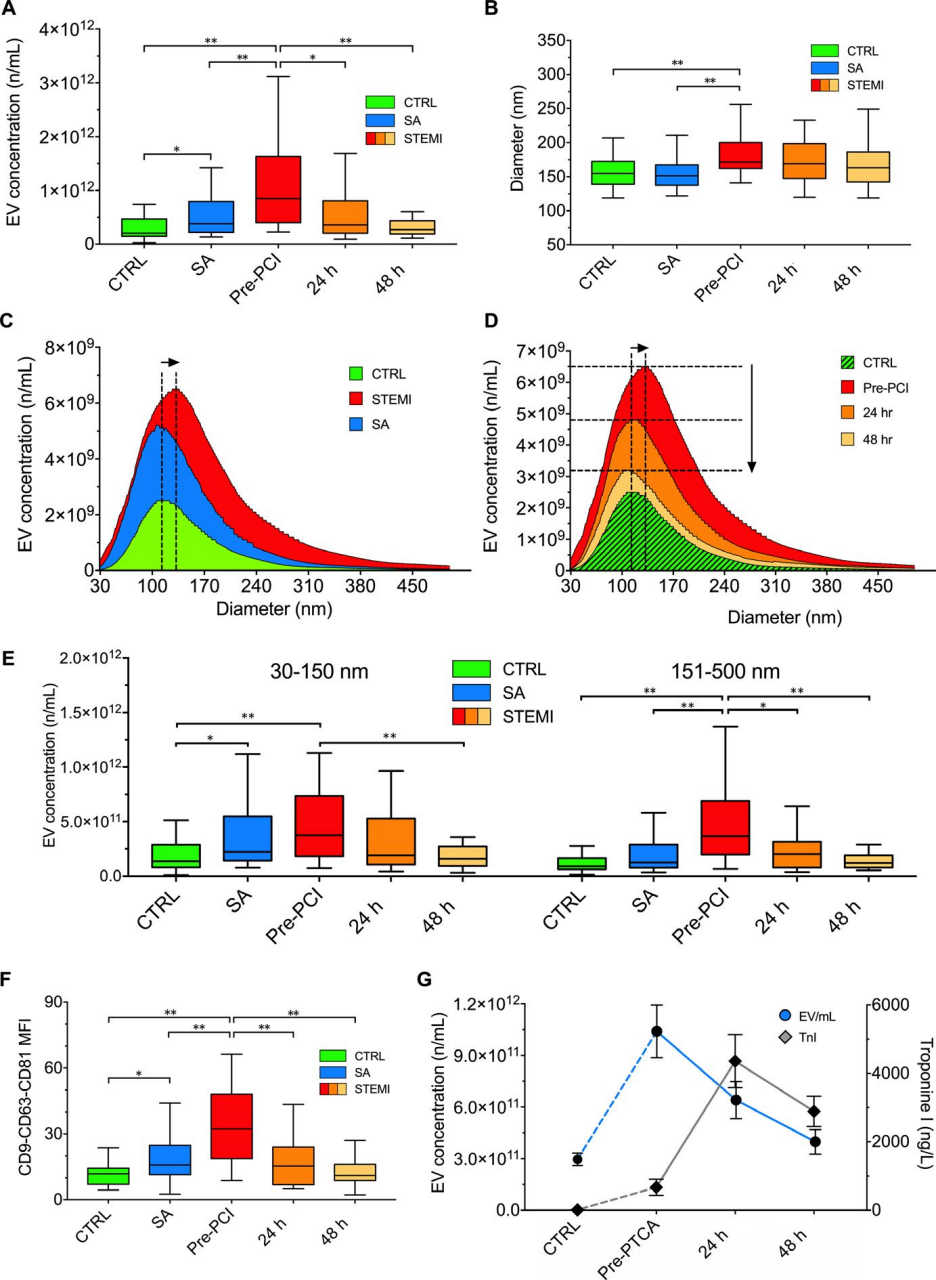
Nanoparticle tracking analysis. Nanoparticle Tracking Analysis of circulating EVs in the three groups of patients of the training cohort (controls, CTRL vs stable angina, SA *vs*. STEMI pre‐PCI and 24/48 h thereafter). (A) EV (extracellular vesicles) concentration; (B) EV diameter; (C and D) Cumulative distribution plot combining EV concentration (n/mL; y‐axes) and diameter (nm; x‐axes); (E) EV number stratified for EV diameter (30‐150 nm vs 151‐500 nm). (F) Mean fluorescence intensity (MFI) for CD9, CD63 and CD81 markers measured by flow cytometry. (G) EV concentrations and hs‐troponin in Ctrl and STEMI patients at different time points. Data and statistical analysis: see Tables S7 and S8. Data are shown as median and interquartile range. *P < .05; **P < .01

Serum samples were pre‐cleared by serial centrifugation steps in order to remove intact cells, cellular debris and larger EVs, while enriching in smaller particles. EV number and diameter decreased after each centrifugation step, showing a trend to depletion of particles larger than 250 nm (*P* > .05; Figure S1B). Before performing EV immunocapture, particle size and concentration were determined by NTA. In the training cohort, EV concentration measured by NTA was significantly increased in serum samples from STEMI patients on presentation to the emergency department (Tables S7 and S8; median [interquartile range, IQR] concentration, 8.5e11 [4.0e11;1.6e12] compared with SA patients (3.8e11 [2.2e11;7.9e11]) and controls (2.1e11 [1.5e11;4.7e11], *P* < .001; Figure 1A,C,D). In STEMI patients, EV concentration declined at 24 hours and 48 hours. A significant difference in EV concentration was also found between SA patients and controls (*P* < .05). Average EV size was similarly increased in STEMI patients, and to a lesser extent in SA patients (Figure 1B), as evidenced by a higher normalized area under the curve (AUC) for cumulative distribution curves (*P* < .001; Figure 1C,D). After stratification for particle diameter, STEMI patients on presentation to the emergency department showed an increase in both small (30‐150 nm in diameter) and larger EVs (151‐500 nm; *P* < .01 vs. controls), whereas SA patients exhibited an increase in small particles only (*P* < .05; Figure 1E). The time course of the increase in EV concentration and hs‐troponin in the serum of STEMI patients is shown on Figure 1G. Particle concentration peaked on presentation to the emergency department and declined by 24 hours and 48 hours, whereas hs‐troponin peaked at 24 hours.

### 3.3 EV characterization and assay validation

The specificity of EV immuno‐capture assay was assessed by Western blot and correlation analysis with NTA data. Presence of the exosomal markers TSG101 and CD81 after incubation with capture beads was demonstrated by Western blotting (Figure S1D,E). Apolipoproteins A1 and B48 were also detectable in these preparations, as expected, being reduced by up to 90% compared with the respective serum samples, suggesting a negligible lipoprotein contamination. It should be noted, however, that the assay used for FC analysis, selectively measured EV surface epitopes of interest labelled with EV‐specific markers (CD9, CD63 and CD81, tetraspanins generally accepted as EV surface markers), providing for an additional level of EV selectivity (Figure S1). In addition, the expression levels of tetraspanins at FC analysis were directly correlated with EV concentration measured by NTA (R = 0.558; *P* < .001; Figure S1C). As expected, CD9/CD63/CD81 MFI as a measure of EV concentration was also increased in serum samples from STEMI patients compared with SA patients and controls (*P* < .001; Figure 1F). Separate experiments were performed to evaluate whether different protocols for EV isolation may affect expression EV surface antigens. After ultracentrifugation, we found a similar profile as compared with the standard immuno‐capture procedure described above, whereas the supernatant was relatively EV‐depleted, as expected (Figure S2A). Similarly, EV isolation by SEC did not substantially affect EV marker profiles (Figure S2B). No difference was found comparing serum and plasma samples from the same patients (Figure S2C). EV marker profiles measured using 1‐hour incubation of serum supernatant with capture beads were similar to those measured using overnight incubation (Figure S2D). The low internal variability of our protocol was confirmed by analysing twice the same samples (Figure S4).

### 3.4 EV surface epitopes

**FIGURE 2:**
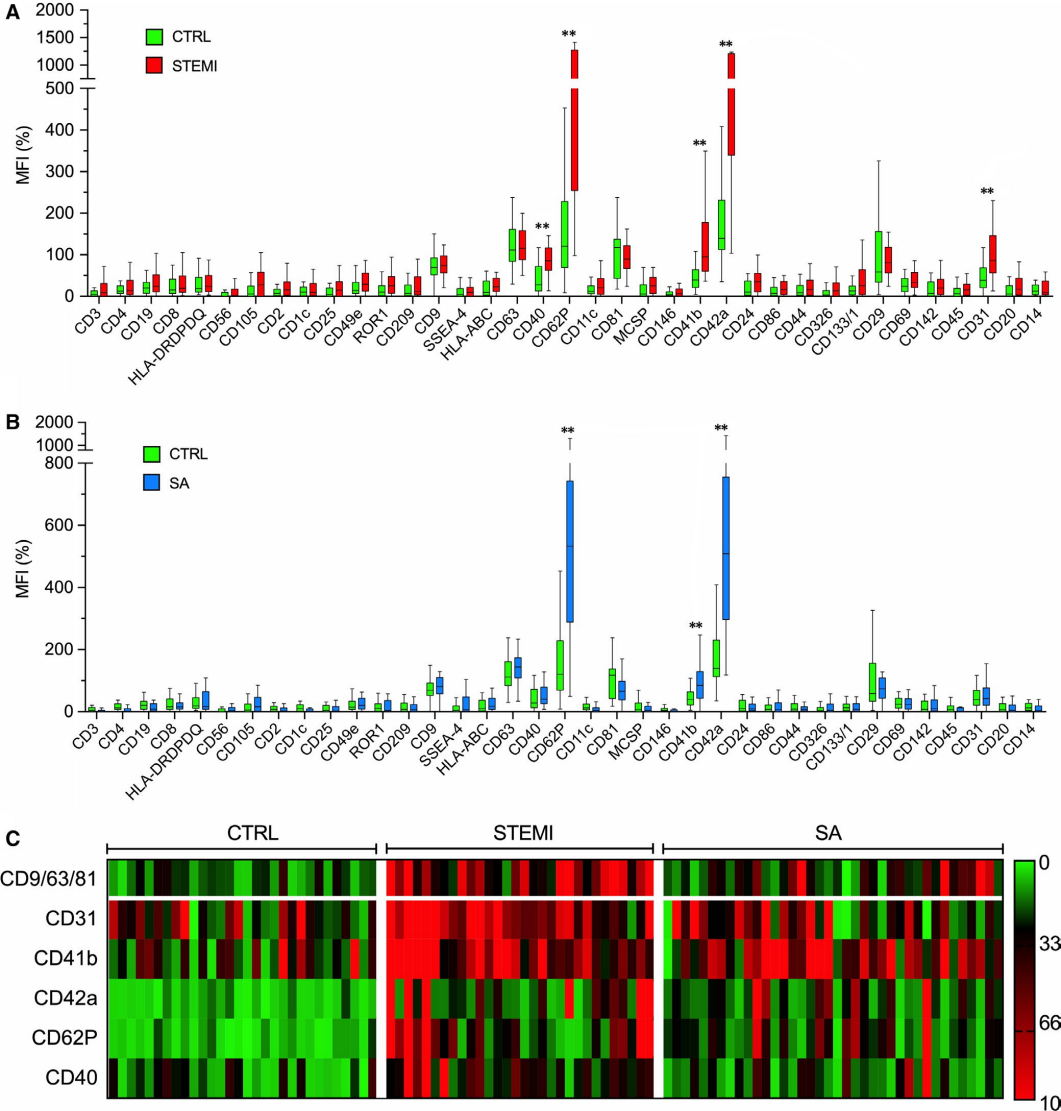
Flow cytometric analysis of EV surface epitopes. Multiplex flow cytometry analysis. Median fluorescence intensities (MFI; [%]) for all EV epitopes, referenced to mean MFI of EV‐specific markers (CD9, CD63 and CD81) in the training cohort. A, STEMI vs Ctrl. B, SA vs Ctrl. C, Heat map showing MFI for EV epitopes expressed at significantly higher levels in STEMI patients vs SA vs controls. Data and statistical analysis: see Table S9. Data are shown as median and interquartile range. *P < .05; **P < .01

**FIGURE 3:**
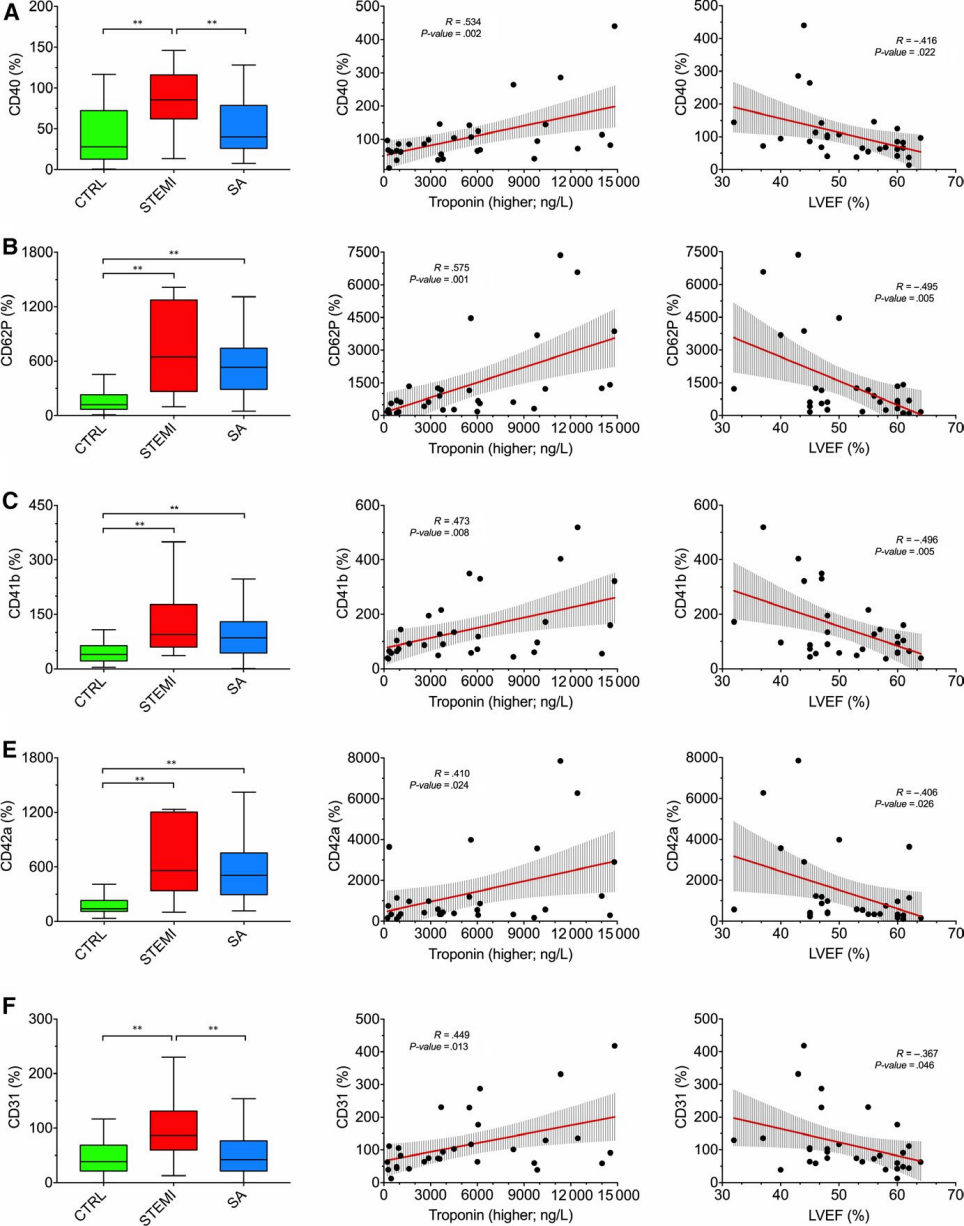
Correlations of EV markers to clinical parameters. Correlation of EV surface epitopes with clinical parameters in the training cohort. Left column: MFI (%) for the indicated EV epitopes, referenced to mean MFI for EV‐specific markers (CD9, CD63, CD81) in different groups (bar graphs). Mid and right columns: correlations of MFI for EV epitopes to hs troponin and LVEF, respectively, in STEMI patients. Regression lines and 95% confidence intervals are shown. *P < .05; **P < .01

EV surface profiling was performed by FC analysis after bead‐based immuno‐capture according to the protocol shown above. EV surface epitope levels in STEMI patients, SA patients and controls are shown in Figure 2 and Tables S9 and S10. In each group, the two most highly expressed EV biomarkers were CD62P (P‐selectin α‐granule membrane protein) and CD42a (platelet membrane glycoprotein), followed by CD41b (platelet membrane glycoprotein II‐b) and CD31 (Platelet‐Endothelial Cell Adhesion Molecule‐1; PECAM‐1). These EV epitopes, along with CD40 (antigen‐presenting cells co‐stimulatory receptor), were increased in STEMI patients on presentation to the emergency department (*P* < .001; Figure 2A). CD62P, CD42a and CD41b levels also were increased in SA patients vs. controls (*P* < .01; Figure 2B), albeit to a lesser extent than in STEMI patients (NS). CD31 and CD40 levels were significantly increased in STEMI patients vs. SA patients (*P* < .01). A heat map of EV surface epitopes in individual participants showed a clear clustering in STEMI patients (Figure 2C). Each of these markers (CD62P, CD42a, CD41b, CD31 and CD40) correlated directly to peak hs‐troponin and inversely to LVEF (Figure 3), which reflect cardiac injury. To assess the potential impact of the biological material, a comparative analysis of EV epitopes using plasma and serum samples from the same subjects was performed in a subset of patients. Each of the five markers that were significantly increased in STEMI patients at pre‐PCI evaluation compared with controls using serum samples, remained significantly increased in STEMI patients using plasma samples (Figure S3). Thus, the results of this analysis in a subset of patients suggest no major impact of the choice of the biological fluid on EV marker analysis. Finally, these five markers all declined over time in STEMI patients, whereas SSEA‐4 (Stage‐Specific‐Embryonic Antigen‐4) was not increased in these patients on presentation to the emergency department, whereas it was increased at 24 hours and 48 hours (*P* = .027 and *P* = .035, respectively; Figure S5).

### 3.5 Performance of EV markers in STEMI discrimination

**FIGURE 4:**
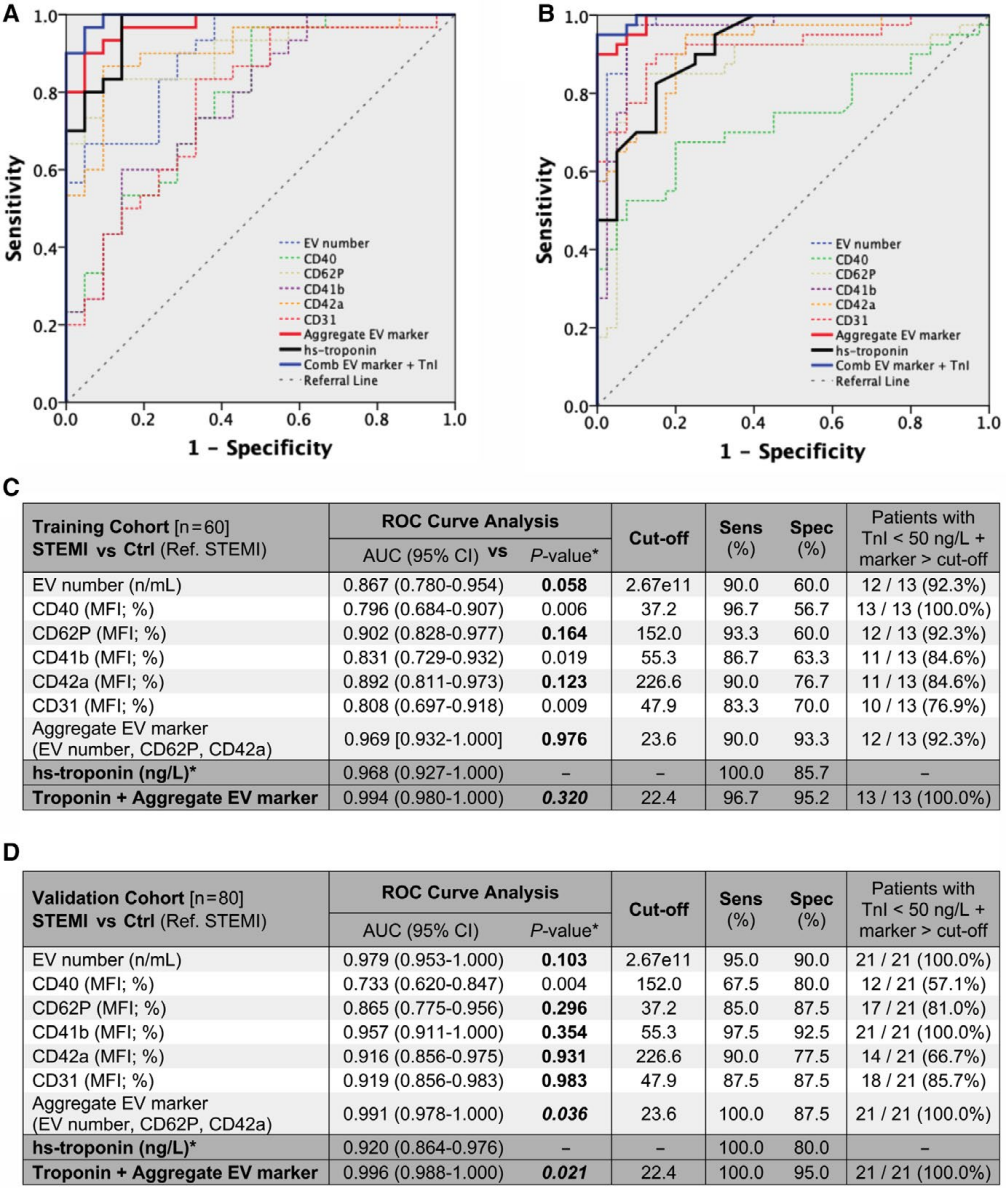
Diagnostic performance of EV markers. Diagnostic performance of EV concentration (by NTA) and five EV markers, compared with hs‐troponin (*P‐values refer to the comparisons of the areas under the curves, AUCs) in the training cohort and in the validation cohort (n = 60 and n = 80, respectively; STEMI patients pre‐PCI *vs*. Ctrl). A and B, ROC curve analysis for hs‐troponin (black curve) compared to individual EV markers (dashed curves), an aggregate EV marker including EV concentration, CD62P MFI, and CD42a MFI; red curve), and to the combination of the aggregate EV marker with hs‐troponin (blue curve). C and D, AUC (95% Confidence Interval; CI), best cut‐off (according to Youden's index analysis), sensitivity (%) and specificity (%), and percentages of patients with minimally increased hs‐troponin (troponin <50 ng/L) showing levels of EV markers higher than the respective cut‐off. Bold characters indicate *P‐*values of diagnostic performances showing non‐inferiority to hs‐troponin (AUC for EV markers < AUC for hs‐troponin; *P* ≥ .05) or superiority to hs‐troponin (AUC for EV markers > AUC for hs‐troponin; *P* < .05)

The diagnostic performance of EV concentration and EV surface markers in discriminating STEMI patients and controls was assessed by multivariate logistic regression and ROC curve analyses. EV concentration and levels of CD62P, CD42a, CD41b, CD40 and CD31 were independent significant predictors of STEMI (Table 2). In the training cohort, ROC curves indicated a high sensitivity for these markers. An aggregate marker including the three most highly discriminating parameters (EV concentration, CD62P levels and CD42a levels) achieved a sensitivity of 90.0% and a specificity of 93.3% (Figure 4A‐C). The evaluation of the AUC confirmed excellent diagnostic performances for these markers (EV concentration, CD62P, CD42a, CD41b, CD40 and CD31). EV concentration, CD62P, CD42a and the aggregate EV marker were not inferior to hs‐troponin (Figure 4C). Of note, the aggregate EV marker was increased above its cut‐off value in 92.3% of STEMI patients presenting with minimally elevated hs‐troponin < 50 ng/L (a cut‐off equalling mean + 1.95*SD of Ctrl values). There also was a trend for a higher AUC for the combination of the aggregate EV marker and hs‐troponin (0.994; 95% CI: 0.980‐1.000) compared with hs‐troponin alone (0.968; CI: 0.927‐1.000; Figure 4C).

**Table 2:** Multivariate logistic regression analysis of EV markers and STEMI diagnosis

STEMI vs Ctrl Ref. STEMI [n = 60]	Age (y)	BMI (kg/m^2^)	Sex (ref. male)	Hypertension (ref. yes)	Diabetes (ref. yes)	Dyslipidemia (ref. yes)	EV marker
EV concentration	1.07 (0.97‐1.19) *P = *.193	1.09 (0.81‐1.48) *P = *.567	6.13 (1.20‐9.02) *P = *.036	1.10 (0.13‐9.06) *P = *.928	9.73 (0.26‐21.60) *P = *.200	2.71 (0.36‐20.30) *P = *.331	1.02 (1.01‐1.03) *P = *.010
CD40 (%)	1.03 (0.96‐1.12) *P = *.393	0.98 (0.77‐1.26) *P = *.894	11.10 (1.34‐27.91) *P = *.026	1.27 (0.21‐7.58) *P = *.792	1.26 (0.04‐21.91) *P = *.898	1.37 (0.24‐7.86) *P = *.724	1.05 (1.02‐1.09) *P = *.002
CD62P (%)	1.01 (0.91‐1.11) *P = *.951	1.03 (0.79‐1.35) *P = *.811	2.59 (0.35‐18.87) *P = *.348	2.13 (0.28‐16.13) *P = *.464	9.63 (0.02‐29.01) *P = *.380	3.56 (0.39‐32.21) *P = *.259	1.01 (1.01‐1.03) *P = *.014
CD41b (%)	1.01 (0.94‐1.07) *P = *.932	1.01 (0.83‐1.22) *P = *.963	5.18 (0.95‐28.57) *P = *.058	1.17 (0.24‐5.76) *P = *.850	1.33 (0.08‐20.83) *P = *.838	1.40 (0.28‐7.02) *P = *.687	1.02 (1.01‐1.03) *P = *.029
CD42a (%)	1.01 (0.92‐1.11) *P = *.849	1.12 (0.87‐1.45) *P = *.383	4.39 (0.51‐37.04) *P = *.177	1.49 (0.19‐11.94) *P = *.709	1.67 (0.07‐34.02) *P = *.752	2.17 (0.28‐16.90) *P = *.459	1.01 (1.01‐1.02) *P = *.007
CD31 (%)	1.05 (0.97‐1.13) *P = *.208	1.03 (0.83‐1.29) *P = *.786	7.75 (1.18‐41.63) *P = *.033	1.34 (0.25‐7.21) *P = *.731	1.55 (0.05‐53.08) *P = *.808	2.10 (0.38‐11.49) *P = *.391	1.05 (1.01‐1.08) *P = *.003
Aggregate EV marker	1.16 (0.80‐1.67) *P = *.428	1.22 (0.04‐1.19) *P = *.079	18.21 (0.05‐56.32) *P = *.362	1.10 (0.13‐9.06) *P = *.928	23.61 (0.02‐64.31) *P = *.164	6.25 (0.01‐37.93) *P = *.812	2.20 (1.04‐4.64) *P = *.038

Note

Association of EV markers and conventional cardiovascular risk factors (including age, BMI, sex, hypertension, diabetes and dyslipidemia), with STEMI diagnosis. Serum samples from STEMI patients on presentation to the emergency department were compared with healthy controls (Ctrl) in the training cohort (n = 60). Odds ratios (95%‐confidence intervals) are shown. Differences were considered significant when *P* < .05.

In the validation cohort, EV concentration, CD62P, CD41b, CD42a and CD31 were not inferior to hs‐troponin, with AUC ranging from 0.865 to 0.979, and sensitivity ranging from 85.0% to 97.5%. The aggregate EV marker achieved higher AUC and diagnostic performance compared with hs‐troponin (*P* = .036), with a sensitivity/specificity of 100.0%/87.5% (Figure 4B‐D). This marker was increased in all STEMI patients presenting with minimally elevated hs‐troponin levels. The combination of the aggregate EV marker and hs‐troponin showed a higher AUC compared with hs‐troponin alone, with a sensitivity/specificity of 100.0%/95.0% (*P* = .021; Figure 4D).

### 3.6 Machine learning algorithm model based on expression levels of EV biomarkers

**FIGURE 5:**
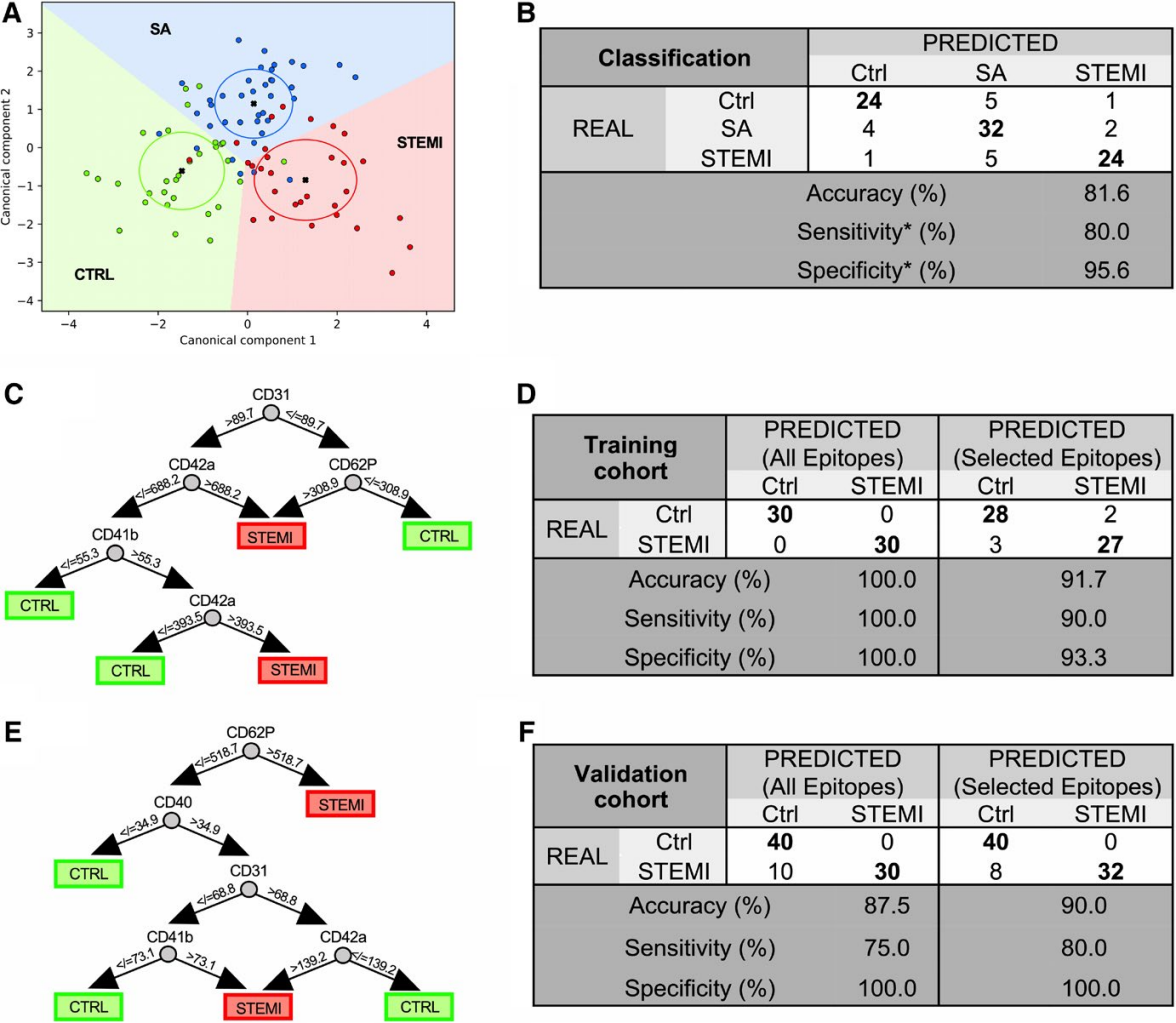
Machine learning diagnostic modelling. Diagnostic performance of machine learning models. A, Canonical plot illustrating patient distribution according to their diagnosis and to the linear weighted combination of EV surface epitope fluorescence values. Red, blue and green circles indicate individual STEMI, SA and control patients. Crosses indicate mean values of (canonical‐1; canonical‐2) for each category. Ellipses include patients with a linear combination coefficient that falls within the mean ± SD (canonical−1+/SD; canonical‐2 ± SD). B, Confusion matrix reporting real and predicted diagnosis (Ctrl vs. SA vs. STEMI), accuracy, sensitivity and specificity, for the linear discriminant analysis (*sensitivity and specificity were calculated using STEMI diagnosis as referral category). C, Representative classification tree from the RF based on MFI data (All Epitopes model). D and F, Confusion matrix reporting real and predicted diagnosis (STEMI *vs*. Ctrl) for two distinct random forest (RF) models based either on the full panel of 37 EV markers (All Epitopes) or five EV markers significantly in STEMI patients (Selected Epitopes) in STEMI vs. Ctrl. Accuracy, sensitivity and specificity are shown for each model in the training cohort (n = 60) and the validation cohort (n = 80). E, Representative classification tree from the RF (Selected Epitopes model)

The linear combination of all EV surface epitopes is shown in the canonical plot (Figure 5A). The model distinguished STEMI patients, SA patients and controls. At first screening analysis, only 6 of 30 STEMI patients were misclassified (5 of them as SA and 1 as control), resulting in an accuracy of 81.6% (Figure 5B). We then developed a diagnostic model for STEMI based on the 37 EV surface epitopes included in the multiplex using RF classification algorithms (Figure 5C,D). The model correctly classified all patients with 100% sensitivity, specificity and accuracy. In the validation cohort, this RF model achieved a sensitivity/specificity of 75.0%/100.0%. A distinct RF model including only the five markers (CD62P, CD42a, CD41b, CD40 and CD31) significantly increased in STEMI patients achieved 91.7% and 90.0% accuracy in the training and validation cohorts, respectively (overfitting bias: 1.7%). This model achieved a sensitivity/specificity of 80.0%/100.0% in the validation cohort (Figure 5E,F).

## 4 DISCUSSION

We have measured multiple EV biomarkers representative of various cell types of origin in the blood of patients with acute MI or chronic CAD presenting with SA. STEMI patients on presentation to the emergency department and to a lesser extent SA patient showed an increase in circulating EV numbers, compared to healthy individuals. Both STEMI patients and SA patients showed an increase in small‐size EVs (≤150 nm, the size traditionally associated with exosomes), whereas only STEMI patients exhibited augmented numbers of medium/large‐sized EVs (151‐500 nm, the size traditionally associated with microvesicles). These results are consistent with the notion that release of microvesicles is induced by cellular stress,^31^ which dramatically increases as a result of acute coronary artery occlusion leading to cardiac injury and the inflammatory response to it. It should be noted, however, that exosomes and microvesicles cannot be precisely discriminated by size.^10^

The two most highly enriched EV markers in STEMI patients, and to a lesser extent in SA patients, were CD62P, a marker of platelet activation, and CD42a, another platelet‐associated marker.^32^ Two additional platelet‐associated markers, CD41b and CD31 (the latter also being expressed by activated endothelial cells), along with CD40, which may reflect immune activation, were increased in STEMI patients. Each of the above markers correlated with STEMI diagnosis at multivariate regression analysis. They also correlated positively to peak hs‐troponin levels and inversely to LVEF, suggesting a direct correlation to injury severity. These findings are in general agreement with previous data showing increased numbers of platelet‐ and endothelial‐derived EVs in patients with ACS, possibly reflecting the formation of coronary thrombotic occlusions, compared to patients with chronic CAD and healthy individuals.^12^, ^13^, ^14^, ^15^, ^16^, ^17^, ^18^, ^19^, ^20^, ^21^, ^22^, ^23^ We also found an increase in SSEA‐4, an antigen expressed by ‘very‐small embryonic‐like stem cells’ that are mobilized from bone marrow after cardiac ischemia and participate in endothelial repair,^33^ at 24‐48 hours in STEMI patients.

We then looked at the potential diagnostic performance of the above EV markers for STEMI. ROC curve analysis revealed a high performance for CD62P, CD42a, CD41b, CD40 and CD31, with CD62P and CD42a showing non‐inferiority to hs‐troponin. We also aggregated the three most discriminating markers (EV concentration, CD62P and CD42a) into a single marker, which achieved 90.0% sensitivity and 93.3% specificity for the diagnosis of STEMI, with non‐inferiority to hs‐troponin, in the training cohort. The aggregate EV marker achieved 100.0% sensitivity at validation. This marker was increased in all STEMI patients with minimally increased hs‐troponin (<50 ng/L) on presentation to the emergency department. Finally, a RF model including the full panel of 37 EV surface epitopes, and a distinct RF model including the five EV markers that were significantly increased in STEMI patients, discriminated these patients and controls with high accuracy, as confirmed in the validation cohort.

Unlike previous studies of EV markers in ACS, which focused on single markers, or a few cell type‐specific markers, we characterized, for the first time, a comprehensive panel of markers, which allowed us to identify those markers that achieved highest diagnostic accuracy in STEMI patients. By combining three markers, diagnostic accuracy was higher than that of individual markers and comparable to cardiac‐specific hs‐troponin I. The power of EV biomarkers derives from the enrichment of potential protein markers which otherwise constitute only a very small proportion of the total proteome of body fluids.^9^ While hs‐troponin has improved the diagnostic performance of cardiac troponin as a marker for ACS, its sensitivity at early stages of myocardial injury is still limited. Our data indicate that EV derived biomarkers are increased at early stages of acute MI, at least in part due to thrombus formation and ischemia‐induced stress to cardiac cells that are still alive, whereas the release of troponin reflects cardiomyocyte death. Thus, EV biomarkers reflect very early stages of cell stress that precede sarcomeric disruption and the release of contractile protein. Potential advantages and disadvantages of EV profiling *versus* cardiac‐specific hs‐troponin in the diagnosis of patients with acute MI are summarized in Table S1. Obviously, the question about the best marker is not the only relevant question. Another question is whether a combination of markers may provide an advantage over single markers. An important example is the combination of copeptin, an acute endogenous stress neuropeptide,^34^ and troponin. While copeptin is non‐specific to myocardial injury, it responds to an immediate neural trigger with concentrations rising early and decreasing gradually over several hours. Adding copeptin to cardiac troponin improved the sensitivity and negative predictive value for the diagnosis of NSTEMI compared to troponin alone.^35^ A similar effect might apply to EV markers, as the combination of the aggregate EV marker and hs‐troponin showed a higher AUC compared with hs‐troponin alone, with a sensitivity/specificity of 100.0%/95.0% for the diagnosis of STEMI (vs. 100.0%/80.0% for troponin alone). These data suggest the possibility that EV markers may play a contributory role in the diagnosis of MI.

Clearly, a technological implementation is still required before clinical applications can be envisaged. In this regard, it is worth noting that impressive progress has recently been scored in ultrasensitive detection of circulating exosomes with microfluidic chips. As an example, a 3D‐nanopatterned microfluidic chip allowed for the detection, in 2‐μL plasma samples from ovarian cancer patients, of exosome subpopulations expressing CD24, EpCAM and FR‐α proteins as potential biomarkers for ovarian cancer.^36^

EV biomarker profiling is emerging as an important general approach for precision medicine and personalized treatment. This approach could be particularly valuable in the critically ill patient, who may show a non‐specific increase of hs‐troponin.^37^ Another example could be represented by patients undergoing cardiac or non‐cardiac surgery, owing to the increased risk of periprocedural cardiac ischemia.^38^ A recent study in patients undergoing cardiac surgery with cardiopulmonary bypass showed a progressive increase in the number of circulating exosomes collected at 2 hours, 4 hours and 24 hours after the onset of the cardiopulmonary bypass.^39^ Another study similarly showed a progressive increase in the plasma concentrations of exosomes in patients undergoing coronary artery bypass graft (CABG). Of note, the increase in circulating exosomes was positively and highly correlated with troponin.^40^

Finally, it should be mentioned that, beyond their diagnostic role, EV‐based biomarkers have predictive value for the prognosis of cardiovascular disease.^41^

Regarding methodological issues, we confirmed the technical reproducibility of the flow cytometric assay used in the present study. We also found no major differences between sample ultracentrifugation and SEC, and between 1‐hour or overnight incubation of serum supernatant with capture beads. These observations support the absence of major methodological confounding factors. Both plasma and serum have been used in previous studies; while biobanking of plasma may be preferable for studies involving isolation of EV RNA, serum also has appropriate uses.^42^ Platelets can be activated to a varying extent during plasma collection, whereas serum sampling involves in vitro platelet activation under standardized conditions. We compared serum and plasma samples in a small subset of patients, observing a similar EV marker profile. In particular, the five EV markers that were significantly increased in serum samples from STEMI patients compared to controls also were significantly increased in plasma. A methodological discussion regarding the more appropriate biological fluid is beyond the scope of this study; anyway, the results of our comparative analysis in a subset of patients suggest that serum samples can be used.

A few study limitations need to be addressed. Because of the proof‐of‐principle character of the study, we decided to investigate STEMI patients, who exhibit comparatively a clear clinical phenotype and hypothetical large changes in circulating EV. The evaluation of unstable angina or NSTEMI patients could be object of future studies. Though the study cohorts were small, accurate diagnostic models could be developed and validated in larger independent populations. Moreover, while healthy individuals were suitable controls for this pilot study, unselected control populations including patients with chronic CAD, chronic inflammatory diseases, thrombotic diseases, cancer and other disorders would more closely reflect daily clinical practice. This point is exemplified by our data, which showed larger differences in EV markers in STEMI patients versus healthy subjects, compared with STEMI patients versus SA patients.

In conclusion, we have shown that circulating EVs in patients with STEMI differ in number, size and surface markers from those in healthy subjects, with SA patients showing somewhat intermediate features. Our findings demonstrate the feasibility of a diagnostic assay for early‐stage acute MI based on EV biomarkers, with a potential usefulness also in chronic CAD patients. The marker profile can be customized to optimize diagnostic accuracy, and the method is amenable to full automation.

## CONFLICT OF INTEREST

The authors confirm that there are no conflicts of interest.

## AUTHOR CONTRIBUTION

**Jacopo Burrello:** Data curation (equal); Formal analysis (equal); Investigation (equal); Writing‐original draft (equal); Writing‐review & editing (equal). **Sara Bolis:** Formal analysis (equal); Methodology (equal); Visualization (equal). **Carolina Balbi:** Formal analysis (equal); Methodology (equal); Visualization (equal). **Alessio Burrello:** Data curation (equal); Formal analysis (equal); Software (equal). **Elena Provasi:** Formal analysis (equal); Methodology (equal); Visualization (equal). **Elena Caporali:** Formal analysis (equal); Investigation (equal); Visualization (equal). **Lorenzo Grazioli Gauthier:** Formal analysis (equal); Investigation (equal); Visualization (equal). **Andrea Peirone:** Formal analysis (equal); Investigation (equal); Visualization (equal). **Fabrizio D'Ascenzo:** Formal analysis (equal); Investigation (equal); Visualization (equal). **Silvia Monticone:** Formal analysis (equal); Investigation (equal); Visualization (equal). **Lucio Barile:** Conceptualization (equal); Funding acquisition (equal); Investigation (equal); Methodology (equal); Visualization (equal). **Giuseppe Vassalli:** Conceptualization (equal); Funding acquisition (equal); Investigation (equal); Writing‐original draft (equal); Writing‐review & editing (equal).

## Supporting information

App S1

## Data Availability

Data that support the findings of this study are available from the corresponding authors upon reasonable request.
