# Supplementary material for: An extracellular vesicle epitope profile is associated with acute myocardial infarction
Source: J Cell Mol Med. 2020 Jul 14;24(17):9945–57. doi: 10.1111/jcmm.15594 (PMC7520329; doi:10.1111/jcmm.15594)
Supplement: Supplementary file 1 — App S1 [file JCMM-24-9945-s001.docx]

**Appendix S1**

**An extracellular vesicle epitope profile is associated**

**with acute myocardial infarction**

Jacopo Burrello^1^, Sara Bolis^1,2^, Carolina Balbi^1^, Alessio Burrello^3^, Elena Provasi^1^,

Elena Caporali^1^, Lorenzo Grazioli Gauthier^1^, Andrea Peirone^4^, Fabrizio D’Ascenzo^4^,

Silvia Monticone^5^, Lucio Barile^2,6,7^, Giuseppe Vassalli^1,6,8^.

(1) Laboratory of Cellular and Molecular Cardiology, and (2) Laboratory for Cardiovascular Theranostics, Cardiocentro Ticino

and Foundation for Cardiovascular Research and Education (FCRE), Lugano, Switzerland;

(3) Department of Electrical, Electronic and Information Engineering "Guglielmo Marconi" (DEI), University of Bologna, Italy;

(4) Department of Medical Sciences, Division of Cardiology, University of Torino, Italy;

(5) Department of Medical Sciences, Division of Internal Medicine, University of Torino, Italy;

(6) Faculty of Biomedical Sciences, Università della Svizzera Italiana (USI), Lugano, Switzerland;

(7) Institute of Life Science, Scuola Superiore Sant’Anna, Pisa, Italy;

(8) Center for Molecular Cardiology, University of Zurich, Switzerland.

**Summary**

Supplementary Figure S1 – Assay validation

Supplementary Figure S2 – Alternate protocols for flow cytometric analysis of EV surface epitopes

Supplementary Figure S3 – Serum- *vs.* plasma- derived EV surface epitopes

Supplementary Figure S4 – Technical reproducibility of MACSPlex human Exosome Kit

Supplementary Figure S5 – EV-surface epitopes from patients with STEMI at different time points

Supplementary Table S1 – EV profiling vs. hs-troponin: advantages and disadvantages

Supplementary Table S2 – EV-surface epitopes analyzed by flow cytometry

Supplementary Table S3 – Characteristics of patients diagnosed with STEMI (training cohort)

Supplementary Table S4 – Pharmacological treatment of included patients (training cohort)

Supplementary Table S5 – Training cohort *vs.* validation cohort

Supplementary Table S6 – Clinical and biochemical characteristics (validation cohort)

Supplementary Table S7 – Nanoparticle Tracking Analysis of CTRL *vs.* STEMI *vs.* SA patients

Supplementary Table S8 – Nanoparticle Tracking Analysis of CTRL *vs.* STEMI (at different time points)

Supplementary Table S9 – Flow Cytometry Analysis of CTRL vs. STEMI vs. SA patients

Supplementary Table S10 – Flow Cytometry Analysis of CTRL vs. STEMI (at different time points)

Extended Methods

***Supplementary Figure S1*** *– Study design and assay validation*

*
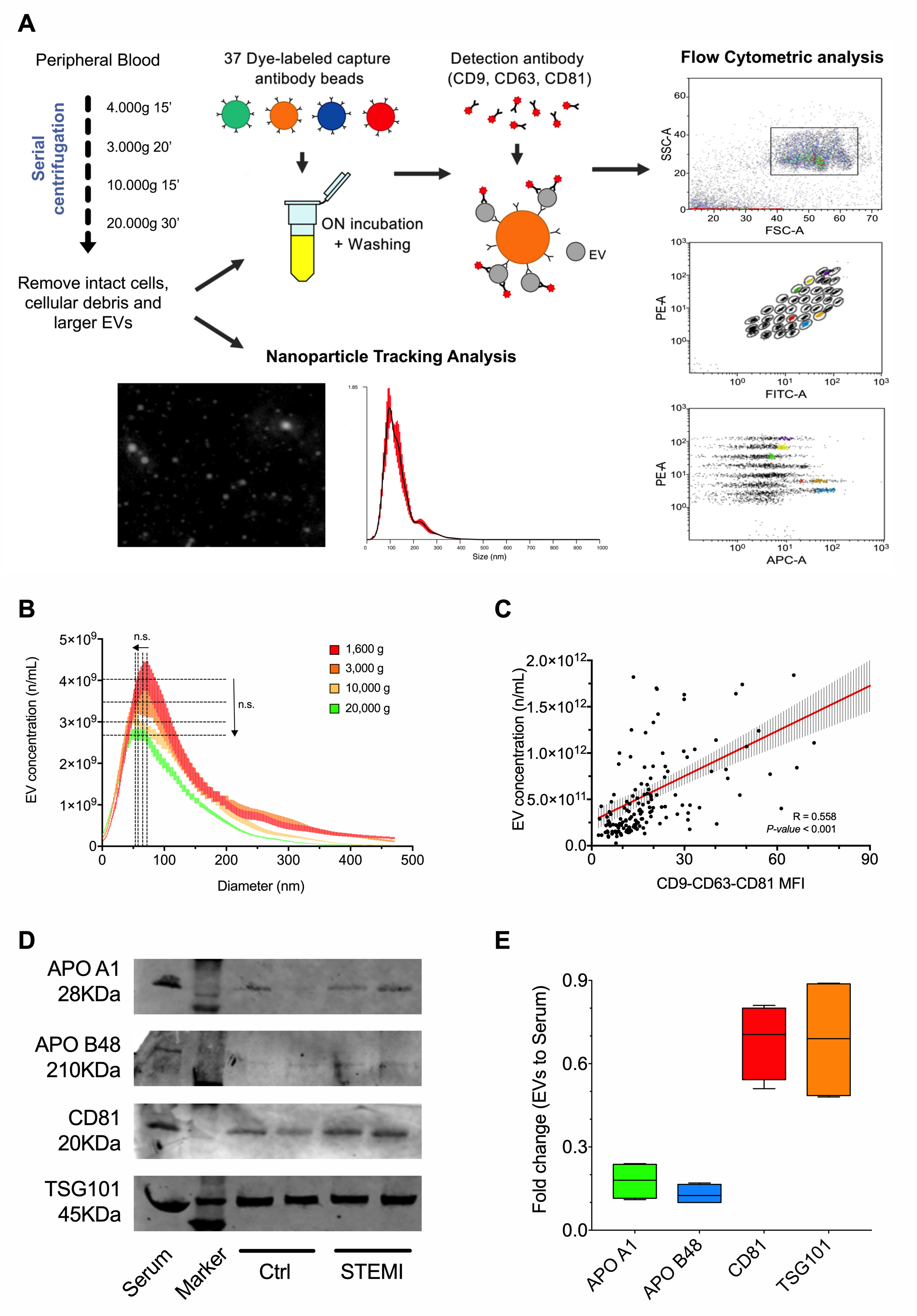
*

(**a**) After serial centrifugations to remove intact cells, cellular debris, and larger extracellular vesicles (EVs), serum was analyzed by nanoparticle tracking analysis or incubated over-night with dye-labeled antibody-coated capture beads and then with detection antibodies against CD9, CD63, and CD81 for 1 hour; analysis was performed by flow cytometry. Modified from Koliha et al.^4^. (**b**) Cumulative distribution plot combining EV concentration (n/mL; y-axes) and diameter (nm; x-axes) after each centrifugation step (n=5; the curves represent mean values ± standard error, dotted lines indicate the curve peak at each step). The arrows indicate the decrease of diameter and number of particles after centrifugation (n.s., not significant reduction). (**c**) Correlation between EV concentration at nanoparticle tracking analysis (y-axis) and CD9-CD63-CD81 median fluorescence intensity (MFI; x-axis) at flow cytometry for patient of the training cohort (n=98); regression lines and 95% confidence intervals are shown. (**d**) Western blot analysis for EV specific markers (CD81 and TSG101) and lipoprotein contaminants (apolipoproteins A1 and B48) in samples from patients with STEMI and controls (n=2 each). Whole serum was compared to EVs after immuno-capture by MACSPlex kit capture beads. (**e**) Histograms showing normalized protein quantification (fold change of protein levels in EVs isolated by beads-immuno-capture *vs.* unprocessed serum samples).

***Supplementary Figure S2****– Alternate protocols for FC analysis of EV surface epitopes*

**
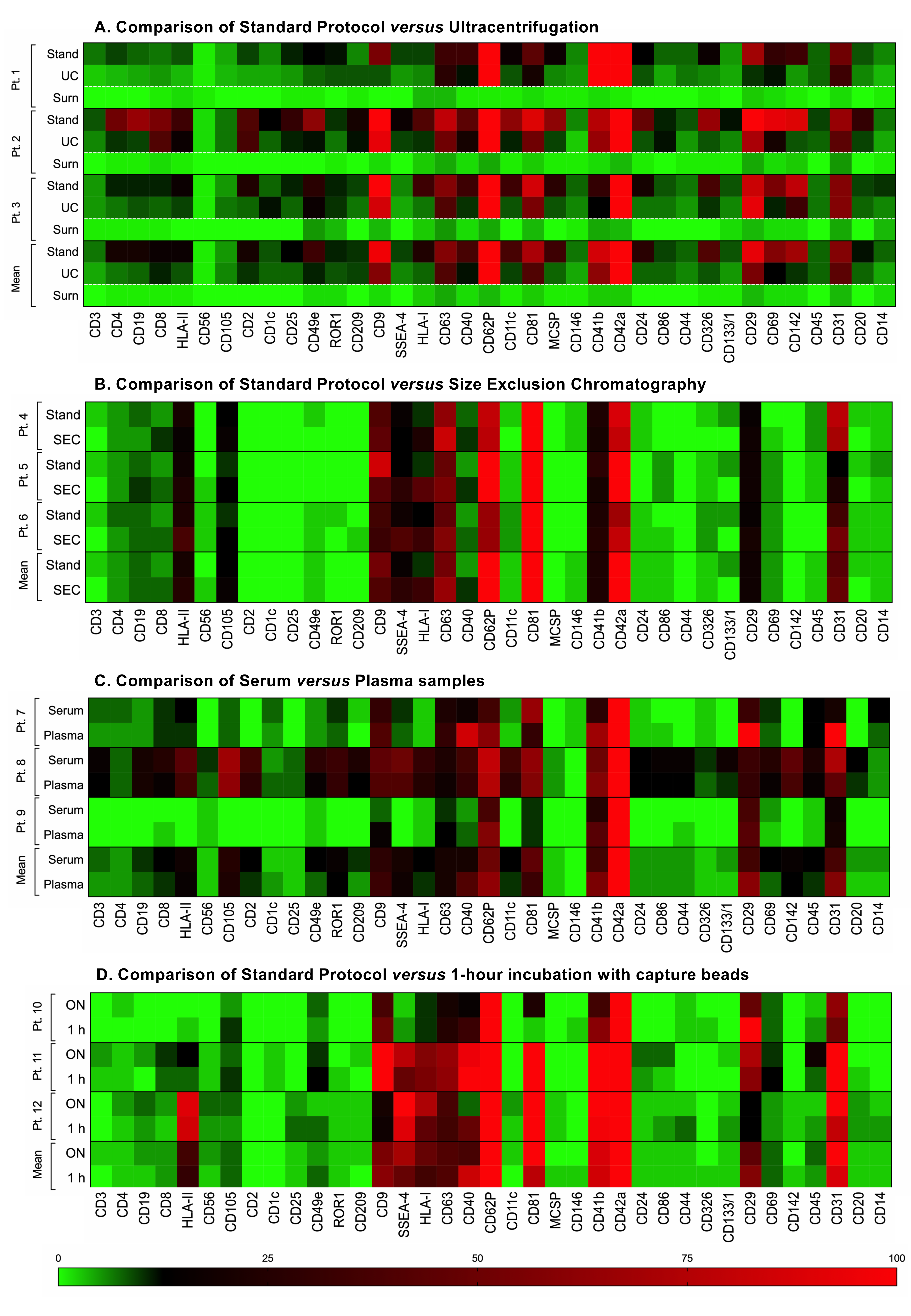
**

Comparison of different protocols for flow cytometric analysis of EV surface epitopes. Heat maps represent the median fluorescence intensity (MFI; red/green, high/low MFI) of each of the 37 EV-surface epitopes measured. (**a**) Serum samples (*n*=3) processed with standard protocol (Stand) compared with ultracentrifugation (UC) protocol. Supernatant after UC (Surn) is shown for comparison purposes. (**b**) Serum samples (*n*=3) processed with standard protocol (Stand) compared with size exclusion chromatography (SEC); (**c**) Serum compared with plasma samples from the same patients (*n*=3) processed with standard protocol. (**d**) Comparison between serum samples (n=3) processed with standard protocol and analyzed after overnight (ON) *vs.* 1-hour incubation with capture beads.

***Supplementary Figure S3*** *– Comparison of serum vs. plasma-derived EV surface epitopes*

*
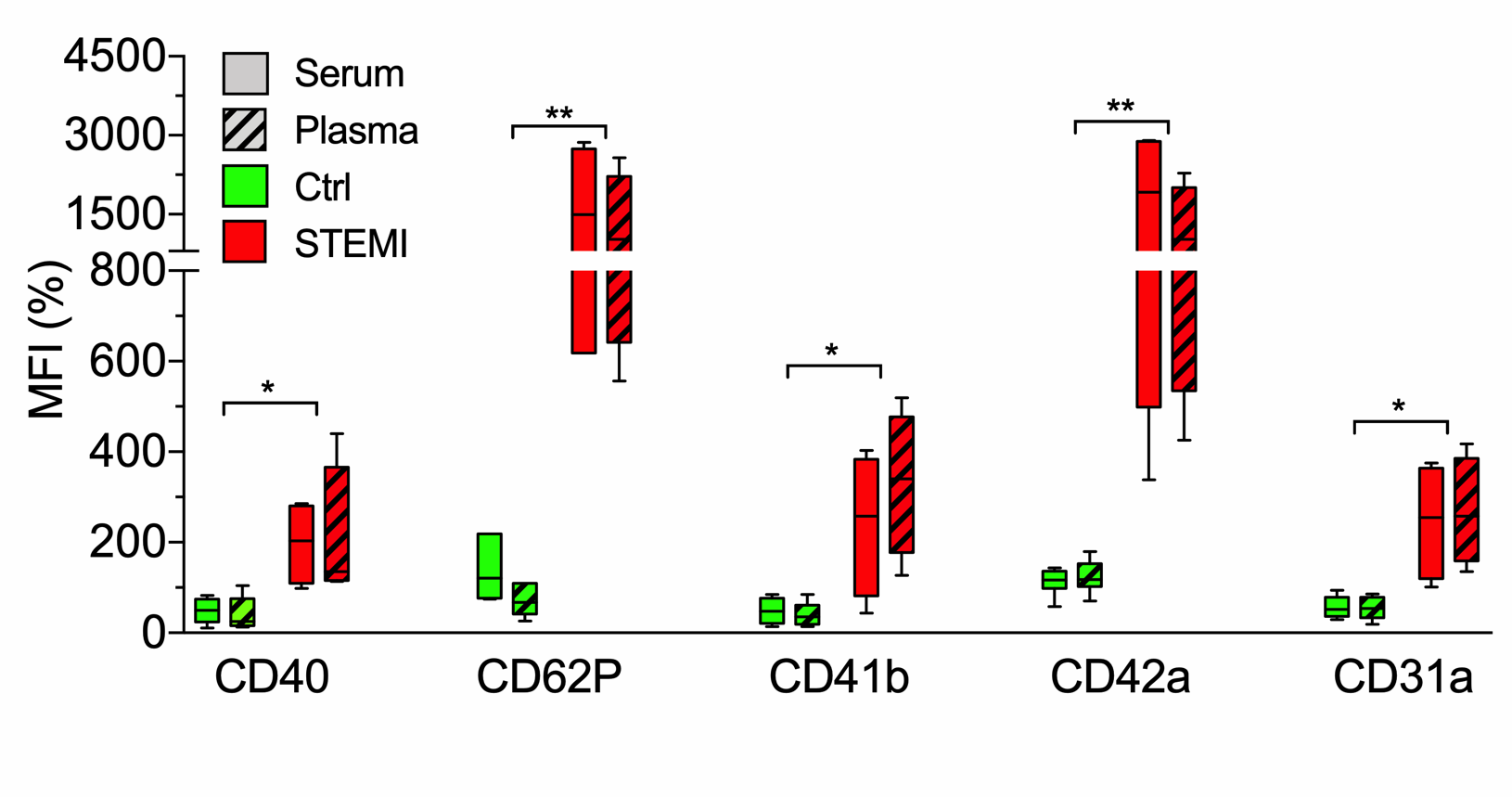
*

A comparative analysis of EV surface epitopes measured in serum samples (filled boxes) and plasma samples (dashed boxes) collected from the same patients was performed to assess a possible impact of the biological material on the results. A randomly selected subset of patients, including STEMI patients at pre-PCI evaluation (red boxes; n=4) and controls (green boxes, n=6), were analyzed. Data are shown for the five surface epitopes that are significantly increased in serum samples of STEMI patients vs. controls. The analysis shows that they also are significantly increased in plasma samples of STEMI patients vs. controls. Data are expressed as median fluorescence intensity (MFI; expressed by percentage) normalized for CD9/CD63/CD81 MFI. Data are shown as median and interquartile range. **p*<0.05; ** *p*<0.01

***Supplementary Figure S4*** *– Technical reproducibility of MACSPlex human Exosome Kit*

*
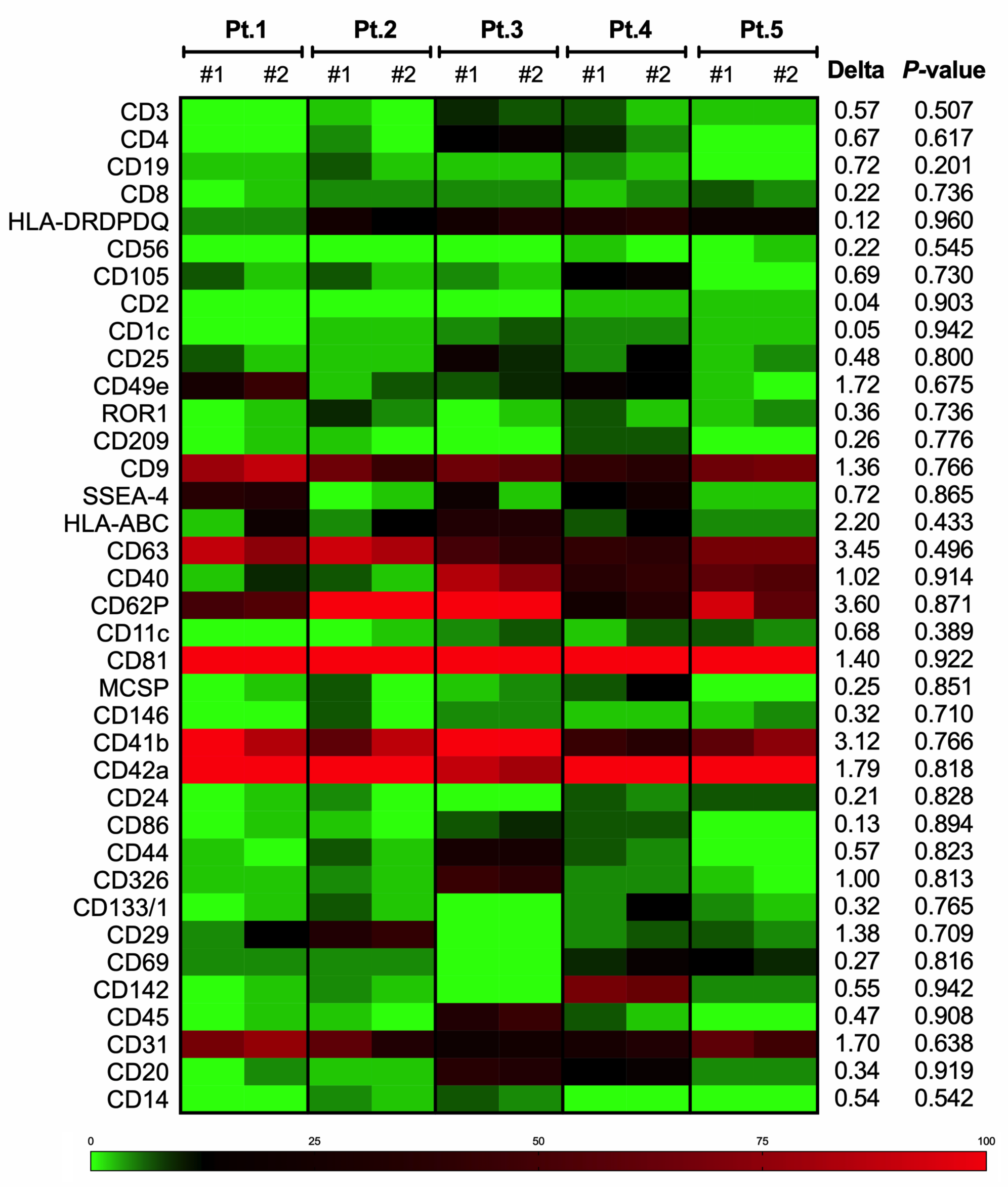
*

The reproducibility of MACSPlex flow cytometric assay was assessed by comparison of duplicates of samples analyzed in different days (internal variability expressed as delta MFI = 0,57%; n=5). The heat map represents the median fluorescence intensity (MFI; red/green, high/low MFI) of each of the 37 EV-surface epitopes measured. *P-*values of less than 0.05 were considered significant.

***Supplementary Figure S5****– EV-surface epitopes from patients with STEMI at different time points*

*
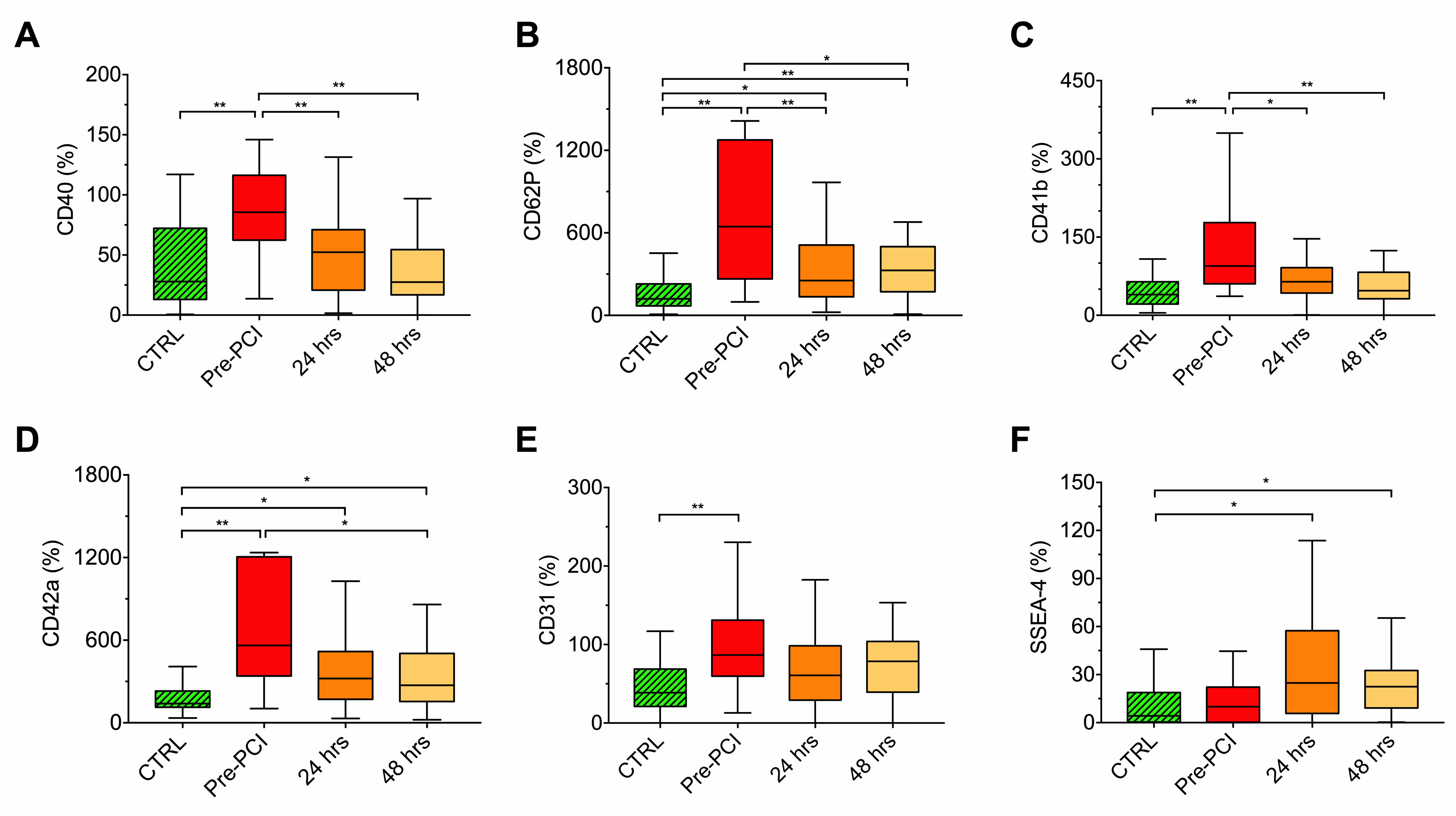
*

Median fluorescence intensity (MFI; expressed by percentage) normalized for CD9/CD63/CD81 MFI, of EV surface epitopes significantly increased in STEMI patients before percutaneous coronary intervention (pre-PCI). (**a**) CD40; (**b**) CD62P; (**c**) CD41b; (**d**) CD42a; (**e**) CD31; (**f**) SSEA-4. STEMI patients were evaluated at pre-PCI (red), at 24 hrs (dark orange), and at 48 hrs (light orange) vs. controls (CTRL; green). Data and statistical analysis: see Table S10. Data are shown as median and interquartile range. **p*<0.05; ** *p*<0.01.

***Supplementary Table S1*** *– EV profiling vs. hs-troponin: advantages and disadvantages*

|  | **EV profiling** | **Cardiac hs-troponin** |
| --- | --- | --- |
| **Advantages** | Early increase in blood  (before necrosis of cardiomyocytes) | Low cost |
|  | Potential  prognostic value | Fast (measurement available  within about 30 minutes) |
|  | Potential hints on thrombus burden | Specific for myocardial damage of various origin |
| **Disadvantages** | Need for protocol standardization and technology implementation | Delayed increase in blood (during necrosis) |
|  | Time-consuming and expensive  if compared to troponin | Blood kinetics depends on renal function |

Potential advantages and disadvantages of EV derived biomarkers as compared to cardiac high-sensitive troponin.

***Supplemental Table S2*** *– EV-surface epitopes analyzed* by flow cytometry

| **EV-surface epitope** | **Description** |
| --- | --- |
| CD3 | T cells transmembrane co-receptor |
| CD4 | T and B cells transmembrane glycoprotein |
| CD19 | Surface molecule co-stimulating B cells activation |
| CD8 | T cells transmembrane glycoprotein |
| HLA-DRDPDQ | Type II - Major Histocompatibility Complex -DR / -DP / -DQ |
| CD56 | Neural Cell Adhesion Molecule |
| CD105 | Endoglin (TGF-beta receptor complex) |
| CD2 | T and NK cells transmembrane glycoprotein |
| CD1c | T cells surface glycoprotein involved in antigen presentation |
| CD25 | Interleukin-2 Receptor alpha-chain |
| CD49e | Integrin alpha-5 |
| ROR1 | Neurotrophic Tyrosine Kinase, receptor-related 1 |
| CD209 | Dendritic Cell-Specific Intercellular adhesion molecule-3 |
| CD9 | Tetraspanine super-family – EVs surface protein |
| SSEA-4 | Stage-Specific Embryonic Antigen-4 |
| HLA-ABC | Type II - Major Histocompatibility Complex -A / -B / -C |
| CD63 | Tetraspanine super-family – EVs surface protein |
| CD40 | Antigen Presenting Cells co-stimulatory receptor |
| CD62P | P-selectin (platelet alpha-granule membrane protein) |
| CD11c | Integrin, alpha-X (complement component 3 receptor 4 subunit) |
| CD81 | Tetraspanine super-family – EVs surface protein |
| MCSP | Melanoma-associated Chondroitin Sulfate Proteoglycan |
| CD146 | Melanoma Cell Adhesion Molecule |
| CD41b | Platelet membrane glycoprotein II-b |
| CD42a | Platelet membrane glycoprotein IX |
| CD24 | Heat stable antigen 24 – cell adhesion molecule |
| CD86 | Antigen Presenting Cells co-stimulatory protein |
| CD44 | Homing Cell Adhesion Molecule or Phagocytic glycoprotein-1 |
| CD326 | Epithelial Cell Adhesion Molecule |
| CD133/1 | Prominin-1 transmembrane glycoprotein |
| CD29 | Integrin beta-1 (extracellular matrix component) |
| CD69 | Transmembrane C-Type Lectin protein |
| CD142 | Platelet Tissue Factor-III, F-3 |
| CD45 | Protein Tyrosine Phosphatase, Receptor type-C |
| CD31 | Platelet-Endothelial Cell Adhesion Molecule-1 |
| CD20 | B-lymphocyte antigen-20, glycosylated phosphor-protein |
| CD14 | Lipopolysaccharide co-receptor binding protein |

Description of the 37 EV-surface epitopes included in the multiplex flow cytometry analysis.

***Supplemental Table S3*** *– Characteristics of patients diagnosed with STEMI (training cohort)*

| **Variable** | **STEMI**  [n=30] |
| --- | --- |
| Time from chest pain onset (hrs) | 2.75 [2.00; 4.00] |
| hs troponin (Pre-PCI; ng/L) | 669 ± 1295.0 |
| hs troponin (24 hrs; ng/L) | 4710 ± 4233.8 |
| hs troponin (48 hrs; ng/L) | 3061 ± 2948.0 |
| hs troponin (higher; ng/L) | 5936 ± 4567.5 |
| Infarcted myocardial region  Anterior  Lateral  Infero-lateral  Infero-posterior  Inferior | 10 (33.3)  5 (16.7)  3 (10.0)  9 (30.0)  3 (10.0) |
| Coronary lesion (n) | 1 [1; 2] |
| Vessel culprit lesion  AIA  DX  Cx | 15 (50.0)  13 (43.3)  2 (6.7) |

Characteristics of patients with ST-segment elevation myocardial infarction (STEMI) from the training cohort. PCI (Percutaneous Coronary Intervention). Data are expressed as median [interquartile range], or mean ± standard deviation, or absolute number (percentage), when appropriated.

***Supplementary Table S4*** *– Pharmacological treatment of included patients (training cohort)*

| **Variable** | **CTRL**  [n=30] | **STEMI**  [n=30] | **SA**  [n=38] | ***P-*value** |
| --- | --- | --- | --- | --- |
|  |  |  |  |  |
| ACEIs (%) | 3 (10.0) | 5 (16.7) | 11 (28.9) | 0.197 |
| ARBs (%) | 5 (16.7) | 6 (20.0) | 9 (23.7) | 0.893 |
| BBs (%) | 3 (10.0) | 4 (13.3) | 12 (31.6) | 0.077 |
| CCBs (%) | 5 (16.7) | 2 (6.7) | 5 (13.2) | 0.371 |
| Diuretics (%) | 3 (10.0) | 4 (13.3) | 10 (26.3) | 0.229 |
| Nitrates (%) | 2 (6.7) | 0 (0.0) | 2 (5.3) | 0.336 |
| Statins (%) | 5 (16.7) | 6 (20.0) | 12 (31.6) | 0.417 |
| Oral hypoglycemic agents (%) | 1 (3.3) | 4 (13.3) | 7 (18.4) | 0.228 |
| ASA (%) | 3 (10.0) | 4 (13.3) | 10 (26.3) | 0.229 |

Therapy at enrollment. Controls (CTRL) were compared to patients with ST-segment elevation myocardial infarction (STEMI), or stable angina (SA), from the training cohort. ACEIs (Angiotensin-Converting Enzyme Inhibitors), ARBs (Angiotensin II Receptor Blockers), BBs (β-Blockers), CCBs (Calcium Channel Blockers), ASA (Acetyl-Salicylic Acid). Data are expressed as absolute number (percentage). *P*-values of less than 0.05 were considered significant.

***Supplementary Table S5*** *– Comparison of the training cohort vs. validation cohort (clinical and biochemical characteristics of STEMI patients and controls)*

| **Variable** | **Training cohort**  [n=60] | **Validation cohort**  [n=80] | ***P-*value** |
| --- | --- | --- | --- |
|  |  |  |  |
| Diagnosis of STEMI (%) | 30 (50.0) | 40 (50.0) | 1.000 |
| Age (years) | 62 ± 11.2 | 64 ± 12.8 | 0.345 |
| Sex (ref. male) | 42 (70.0) | 59 (73.8) | 0.624 |
| Familiarity for CAD (%) | 17 (28.3) | 16 (20.0) | 0.250 |
| Hypertension (%) | 22 (36.7) | 41 (51.2) | 0.086 |
| Diabetes (%) | 5 (8.3) | 9 (11.3) | 0.569 |
| Dyslipidemia (%) | 26 (43.3) | 35 (43.8) | 0.961 |
| CKD (%) | 4 (6.7) | 3 (3.8) | 0.433 |
| Smoking Habit (%) | 6 (10.0) | 10 (12.5) | 0.241 |
| Systolic BP (mmHg) | 132 ± 19.9 | 132 ± 23.3 | 0.991 |
| Diastolic BP (mmHg) | 81 ± 11.6 | 81 ± 11.2 | 0.883 |
| Weight (Kg) | 76 ± 12.8 | 74 ± 9.6 | 0.226 |
| BMI (Kg/m^2^) | 26.3 ± 3.86 | 25.8 ± 2.67 | 0.349 |
| hs troponin (ng/L) | 396 ± 1039.5 | 225 ± 578.1 | 0.228 |
| WBC (n/L) | 8970 ± 2947.4 | 9843 ± 4530.4 | 0.218 |
| Creatinine (mg/dL) | 0.92 ± 0.216 | 0.98 ± 0.224 | 0.117 |
| GFR (mL/min) | 88 ± 27.1 | 83 ± 21.2 | 0.243 |
| CRP (mg/L) | 5.2 ± 6.28 | 6.8 ± 4.78 | 0.112 |
| Glycemia (mmol/L) | 7.5 ± 2.91 | 7.7 ± 2.77 | 0.818 |
| Total Cholesterol (mmol/L) | 4.9 ± 1.24 | 4.8 ± 0.93 | 0.592 |
| HDL (mmol/L) | 1.5 ± 1.34 | 1.2 ± 0.24 | 0.158 |
| Triglycerides (mmol/L) | 1.5 ± 1.70 | 1.1 ± 0.43 | 0.117 |
| LVEF at echo (%) | 56 ± 8.51 | 54 ± 10.0 | 0.189 |

Clinical and biochemical characteristics of STEMI patients and controls from the training cohort (n=60) compared to the validation cohort (n=80). STEMI (ST-segment Elevation Myocardial Infarction), CAD (Coronary Artery Disease), CKD (Chronic Kidney Disease), BP (Blood Pressure), WBC (White Blood Cells), GFR (Glomerular Filtration Rate), CRP (C-Reactive Protein), LVEF (Left Ventricular Ejection Fraction at echocardiography). Data are expressed as mean ± standard deviation, or absolute number (percentage), when appropriated.

***Supplementary Table S6*** *– Clinical and biochemical characteristics (validation cohort)*

| **Variable** | **CTRL**  [n=40] | **STEMI**  [n=40] | ***P-*value** |
| --- | --- | --- | --- |
|  |  |  |  |
| Age (years) | 63 ± 12.6 | 64 ± 13.2 | 0.659 |
| Sex (ref. male) | 29 (72.5) | 30 (75.0) | 0.799 |
| Familiarity for CAD (%) | 7 (17.5) | 9 (22.5) | 0.576 |
| Hypertension (%) | 20 (50.0) | 21 (52.5) | 0.823 |
| Diabetes (%) | 5 (12.5) | 4 (10.0) | 0.723 |
| Dyslipidemia (%) | 17 (42.5) | 18 (45.0) | 0.822 |
| CKD (%) | 1 (2.5) | 2 (5.0) | 0.556 |
| Smoking Habit (%) | 5 (12.5) | 5 (12.5) | 0.754 |
| Systolic BP (mmHg) | 131 ± 20.0 | 134 ± 28.1 | 0.729 |
| Diastolic BP (mmHg) | 83 ± 11.4 | 78 ± 10.6 | 0.270 |
| Weight (Kg) | 73 ± 9.2 | 75 ± 10.0 | 0.522 |
| BMI (Kg/m^2^) | 26.0 ± 2.70 | 25.6 ± 2.67 | 0.497 |
| hs troponin (ng/L) | 9 ± 11.4 | 442 ± 761.9 | **0.001** |
| WBC (n/L) | 6138 ± 1364.0 | 12252.5 ± 4232.2 | **<0.001** |
| Creatinine (mg/dL) | 0.97 ± 0.220 | 0.99 ± 0.229 | 0.638 |
| GFR (mL/min) | 86 ± 23.6 | 81 ± 19.6 | 0.390 |
| CRP (mg/L) | 6.1 ± 4.14 | 7.4 ± 5.25 | 0.250 |
| Glycemia (mmol/L) | 7.2 ± 2.42 | 8.3 ± 3.13 | 0.247 |
| Total Cholesterol (mmol/L) | 4.8 ± 0.87 | 4.8 ± 1.05 | 0.872 |
| HDL (mmol/L) | 1.2 ± 0.25 | 1.2 ± 0.22 | 0.814 |
| Triglycerides (mmol/L) | 1.1 ± 0.45 | 1.1 ± 0.41 | 0.925 |
| LVEF at echo (%) | 60 ± 4.5 | 50 ± 10.4 | **<0.001** |

Clinical and biochemical characteristics of patients with ST-segment elevation myocardial infarction (STEMI; n=40) compared to controls (CTRL; n = 40), from the validation cohort. CAD (Coronary Artery Disease), CKD (Chronic Kidney Disease), BP (Blood Pressure), WBC (White Blood Cells), GFR (Glomerular Filtration Rate), CRP (C-Reactive Protein), LVEF (Left Ventricular Ejection Fraction at echocardiography). Data are expressed as mean ± standard deviation, or absolute number (percentage), when appropriated. *P-*values of less than 0.05 were considered significant and indicated by bold characters.

***Supplemental Table S7*** *– Nanoparticle Tracking Analysis of CTRL vs. STEMI vs. SA patients*

| **Variable** | **CTRL**  [n=30] | **STEMI**  [n=30] | **SA**  [n=38] | **Overall *P-*value** | **Pairwise Comparisons** | | |
| --- | --- | --- | --- | --- | --- | --- | --- |
|  |  |  |  |  | **CTRL *vs.* STEMI** | **CTRL *vs.* SA** | **STEMI *vs.* SA** |
| Diameter  (nm) | 155  [139; 173] | 172  [162; 200] | 152  [138; 167] | **<0.001** | **0.004** | 1.000 | **<0.001** |
| EV concentration (n/mL)  [all vesicles] | 2.07e11  [1.50e11; 4.70e11] | 8.50e11  [4.01e11; 1.63e12] | 3.82e11  [2.23e11; 7.93e11] | **<0.001** | **<0.001** | **0.018** | **0.023** |
| EV concentration (n/mL)  [30-150 nm] | 1.36e11  [8.06e10; 2.89e11] | 3.76e11  [1.84e11; 7.37e11] | 2.25e11  [1.43e11; 5.48e11] | **<0.001** | **<0.001** | **0.012** | 0.437 |
| EV concentration (n/mL)  [151-500 nm] | 9.04e10  [6.33e10; 1.66e11] | 3.68e11  [1.99e11; 6.90e11] | 1.26e11  [7.74e10; 2.89e11] | **<0.001** | **<0.001** | 0.154 | **0.001** |
| Area under  NTA curve | 0.054  [0.037; 0.132] | 0.245  [0.111; 0.478] | 0.105  [0.058; 0.227] | **<0.001** | **<0.001** | **0.018** | **0.022** |
| CD9-CD63-CD81 MFI | 11.84  [7.09; 14.32] | 32.25  [18.74; 48.13] | 15.81  [11.46; 24.75] | **<0.001** | **<0.001** | **0.012** | **0.002** |

Data from Nanoparticle Tracking Analysis (NTA). Controls (CTRL) were compared to patients with ST-segment elevation myocardial infarction (STEMI), or stable angina (SA), from the training cohort. EV (Extracellular Vesicle), MFI (Median Fluorescence Intensity). Data are expressed as median and interquartile range. *P-*values of less than 0.05 were considered significant and indicated by bold characters.

***Supplemental Table S8*** *– Nanoparticle Tracking Analysis of CTRL vs. STEMI (at different time points)*

| **Variable** | **CTRL**  [n=30] | **Pre-PCI**  [n=30] | **24 hrs**  [n=30] | **48 hrs**  [n=30] | **Overall**  ***P-*value** | **Pairwise Comparisons** | | | | | |
| --- | --- | --- | --- | --- | --- | --- | --- | --- | --- | --- | --- |
|  |  |  |  |  |  | **CTRL vs. Pre-PCI** | **CTRL vs. 24 hrs** | **CTRL vs. 48 hrs** | **Pre-PCI vs. 24 hrs** | **Pre-PCI vs. 48 hrs** | **24 hrs vs. 48 hrs** |
| Diameter  (nm) | 155  [139; 173] | 172  [162; 200] | 169  [148; 199] | 163  [142; 187] | **0.017** | **0.011** | 0.274 | 1.000 | 1.000 | 0.454 | 1.000 |
| EV concentration (n/mL)  [all vesicles] | 2.07e11  [1.50e11; 4.70e11] | 8.50e11  [4.01e11; 1.63e12] | 3.60e11  [2.08e11; 8.07e11] | 2.73e11  [1.91e11; 4.37e11] | **<0.001** | **<0.001** | 0.174 | 1.000 | **0.026** | **<0.001** | 1.000 |
| EV concentration (n/mL)  [30-150 nm] | 1.36e11  [8.06e10; 2.89e11] | 3.76e11  [1.84e11; 7.37e11] | 1.92e11  [1.06e11; 5.29e11] | 1.60e11  [9.29e10; 2.72e11] | **<0.001** | **<0.001** | 0.634 | 1.000 | 0.112 | **0.002** | 1.000 |
| EV concentration (n/mL)  [151-500 nm] | 9.04e10  [6.33e10; 1.66e11] | 3.68e11  [1.99e11; 6.90e11] | 2.04e11  [7.91e10; 3.15e11] | 1.21e11  [7.85e10; 1.93e11] | **<0.001** | **<0.001** | **0.032** | 1.000 | **0.048** | **<0.001** | 0.800 |
| Area under  NTA curve | 0.054  [0.037; 0.132] | 0.245  [0.111; 0.478] | 0.099  [0.053; 0.232] | 0.073  [0.048; 0.122] | **<0.001** | **<0.001** | 0.170 | 1.000 | **0.027** | **<0.001** | 1.000 |
| CD9-CD63-CD81 MFI | 11.84  [7.09; 14.32] | 32.25  [18.74; 48.13] | 15.21 [6.89; 23.97] | 11.11  [8.75; 16.11] | **<0.001** | **<0.001** | 0.359 | 1.000 | **0.001** | **<0.001** | 0.909 |

Data from Nanoparticle Tracking Analysis (NTA). Subjects with ST-segment elevation myocardial infarction (STEMI) were compared to controls (CTRL), from the training cohort. STEMI patients were evaluated before percutaneous coronary intervention (pre-PCI) and after 24 or 48 hrs from reperfusion. EV (Extracellular Vesicle), MFI (Median Fluorescence Intensity). Data are expressed as median and interquartile range. *P-*values of less than 0.05 were considered significant and indicated by bold characters.

***Supplemental Table S9*** *– Flow Cytometry Analysis of CTRL vs. STEMI vs. SA patients*

| **Variable**  (MFI; %) | **CTRL**  [n=30] | **STEMI**  [n=30] | **SA**  [n=30] | **Overall *P-*value** | **Pairwise Comparisons** | | |
| --- | --- | --- | --- | --- | --- | --- | --- |
|  |  |  |  |  | **CTRL *vs.* STEMI** | **CTRL *vs.* SA** | **STEMI  *vs.* SA** |
| CD3 | 4.81  [0.84; 13.43] | 8.38  [0.08; 30.99] | 0.79  [0.00; 5.01] | 0.054 | - | - | - |
| CD4 | 12.51  [6.75; 25.30] | 14.39  [3.91; 38.85] | 1.79  [0.00; 10.20] | 0.183 | - | - | - |
| CD19 | 20.29  [7.50; 33.79] | 24.00  [10.82; 52.12] | 8.84  [2.88; 26.45] | 0.160 | - | - | - |
| CD8 | 15.56  [6.77; 41.46] | 19.91  [9.95; 49.05] | 15.17  [8.34; 28.85] | 0.590 | - | - | - |
| HLA-DRDPDQ | 18.72  [10.38; 45.64] | 24.46  [11.41; 50.54] | 16.52  [7.57; 64.57] | 0.830 | - | - | - |
| CD56 | 0.45  [0.00; 9.52] | 2.16  [0.00; 17.49] | 3.30  [0.00; 13.73] | 0.606 | - | - | - |
| CD105 | 5.89  [0.00; 24.51] | 27.93  [0.00; 57.94] | 15.99  [2.09; 45.94] | 0.261 | - | - | - |
| CD2 | 7.50  [2.07; 17.06] | 15.72  [2.51; 34.50] | 2.71  [0.45; 12.16] | 0.418 | - | - | - |
| CD1c | 10.32  [1.52; 22.66] | 10.00  [1.89; 31.00] | 1.91  [0.21; 9.01] | 0.188 | - | - | - |
| CD25 | 4.00  [0.41; 20.16] | 14.75  [0.32; 35.46] | 2.87  [0.26; 16.49] | 0.250 | - | - | - |
| CD49e | 14.28  [7.00; 33.87] | 29.00  [12.75; 55.67] | 20.63  [8.57; 43.27] | 0.194 | - | - | - |
| ROR1 | 10.44  [1.97; 25.67] | 25.13  [7.47; 47.65] | 4.32  [0.99; 35.63] | 0.122 | - | - | - |
| CD209 | 7.67  [2.08; 27.53] | 11.25  [3.75; 47.17] | 6.52  [1.17; 23.37] | 0.262 | - | - | - |
| CD9 | 69.10  [51.99; 92.39] | 74.13  [56.64; 97.87] | 80.80  [57.17; 109.87] | 0.591 | - | - | - |
| SSEA-4 | 4.21  [0.39; 18.77] | 9.95  [0.31; 22.20] | 6.55  [1.78; 47.59] | 0.910 | - | - | - |
| HLA-ABC | 9.66  [2.53; 36.92] | 22.98  [11.71; 43.76] | 17.18  [7.27; 43.28] | 0.155 | - | - | - |
| CD63 | 111.74  [84.15; 161.25] | 116.05  [87.92; 158.25] | 143.74  [108.39; 174.01] | 0.104 | - | - | - |
| CD40 | 27.90  [12.99; 72.22] | 85.60  [62.37; 116.33] | 40.35  [26.07; 78.66] | **<0.001** | **<0.001** | 0.745 | **0.003** |
| CD62P | 120.62  [69.34; 228.33] | 645.05  [265.65; 1275.97] | 533.32  [288.30; 742.86] | **<0.001** | **<0.001** | **<0.001** | 0.770 |
| CD11c | 12.17  [6.23; 25.17] | 20.64  [4.68; 42.96] | 4.27  [0.00; 13.22] | 0.107 | - | - | - |
| CD81 | 117.68  [42.87; 137.76] | 89.63  [66.84; 122.10] | 65.86  [37.94; 97.92] | 0.053 | - | - | - |
| MCSP | 6.03  [0.59; 28.04] | 25.30  [7.00; 45.88] | 4.61  [0.37; 17.09] | 0.237 | - | - | - |
| CD146 | 4.41  [1.77; 10.95] | 6.35  [1.00; 18.33] | 0.98  [0.00; 6.22] | 0.059 | - | - | - |
| CD41b | 39.66  [21.54; 64.34] | 94.56  [60.35; 177.84] | 85.18  [43.68; 129.45] | **<0.001** | **<0.001** | **0.001** | 0.783 |
| CD42a | 139.48  [112.62; 231.07] | 561.26  [339.57; 1205.32] | 508.17  [296.60; 755.23] | **<0.001** | **<0.001** | **<0.001** | 1.000 |
| CD24 | 10.18  [2.71; 36.92] | 35.59  [10.93; 55.25] | 9.16  [3.03; 24.45] | 0.075 | - | - | - |
| CD86 | 7.63  [1.72; 21.73] | 16.50  [4.35; 35.68] | 6.42  [0.52; 28.31] | 0.492 | - | - | - |
| CD44 | 8.59  [2.50; 26.51] | 16.23  [2.56; 39.23] | 7.40  [2.50; 15.91] | 0.222 | - | - | - |
| CD326 | 3.05  [0.97; 13.99] | 13.49  [0.00; 32.47] | 4.70  [0.28; 24.76] | 0.869 | - | - | - |
| CD133/1 | 12.86  [3.41; 24.81] | 26.00  [4.26; 64.06] | 7.65  [1.52; 26.30] | 0.094 | - | - | - |
| CD29 | 59.45  [34.05; 156.13] | 80.91  [56.24; 118.37] | 73.73  [43.52; 108.53] | 0.474 | - | - | - |
| CD69 | 23.95  [11.38; 42.97] | 32.95  [21.16; 57.50] | 23.67  [8.18; 41.96] | 0.056 | - | - | - |
| CD142 | 7.65  [0.98; 34.73] | 20.50  [4.43; 41.03] | 9.65  [2.85; 37.82] | 0.315 | - | - | - |
| CD45 | 6.25  [0.48; 19.00] | 15.07  [2.44; 28.92] | 2.07  [0.00; 12.97] | 0.179 | - | - | - |
| CD31 | 38.67  [21.07; 68.79] | 86.72  [59.75; 131.05] | 42.41  [21.12; 76.81] | **<0.001** | **<0.001** | 1.000 | **0.001** |
| CD20 | 5.96  [0.90; 26.01] | 17.63  [3.00; 43.44] | 4.72  [0.42; 21.95] | 0.098 | - | - | - |
| CD14 | 12.38  [3.94; 27.29] | 8.46  [3.61; 36.73] | 3.30  [0.49; 19.23] | 0.097 | - | - | - |

Data from Flow Cytometry (FC) analysis. Controls (CTRL) were compared to patients with ST-segment elevation myocardial infarction (STEMI), or stable angina (SA), from the training cohort. MFI (Median Fluorescence Intensity). Data are expressed as median and interquartile range. *P-*values of less than 0.05 were considered significant and indicated by bold characters.

***Supplemental Table S10*** *– Flow Cytometry Analysis of CTRL vs. STEMI patients (at different time points)*

| **Variable**  (MFI; %) | **CTRL**  [n=30] | **Pre-PCI**  [n=30] | **24 hrs**  [n=30] | **48 hrs**  [n=30] | **Overall**  ***P-*value** | **Pairwise Comparisons** | | | | | |
| --- | --- | --- | --- | --- | --- | --- | --- | --- | --- | --- | --- |
|  |  |  |  |  |  | **CTRL vs. Pre-PCI** | **CTRL vs. 24 hrs** | **CTRL vs. 48 hrs** | **Pre-PCI vs. 24 hrs** | **Pre-PCI vs. 48 hrs** | **24 hrs vs. 48 hrs** |
| CD3 | 4.81  [0.84; 13.43] | 8.38  [0.08; 30.99] | 5.49  [1.75; 26.95] | 6.14  [0.74; 26.90] | 0.923 | - | - | - | - | - | - |
| CD4 | 12.51  [6.75; 25.30] | 14.39  [3.91; 38.85] | 18.38  [3.93; 41.99] | 8.53  [0.82; 36.70] | 0.960 | - | - | - | - | - | - |
| CD19 | 20.29  [7.50; 33.79] | 24.00  [10.82; 52.12] | 29.15  [4.56; 51.89] | 18.90  [2.48; 45.42] | 0.697 | - | - | - | - | - | - |
| CD8 | 15.56  [6.77; 41.46] | 19.91  [9.95; 49.05] | 20.48  [6.89; 43.11] | 13.24  [3.83; 36.81] | 0.549 | - | - | - | - | - | - |
| HLA-DRDPDQ | 18.72  [10.38; 45.64] | 24.46  [11.41; 50.54] | 24.47  [9.68; 46.02] | 22.05  [6.99; 45.72] | 0.923 | - | - | - | - | - | - |
| CD56 | 0.45  [0.00; 9.52] | 2.16  [0.00; 17.49] | 2.83  [0.00; 13.94] | 1.00  [0.00; 7.71] | 0.986 | - | - | - | - | - | - |
| CD105 | 5.89  [0.00; 24.51] | 27.93  [0.00; 57.94] | 10.96  [0.00; 49.70] | 12.74  [0.41; 45.04] | 0.531 | - | - | - | - | - | - |
| CD2 | 7.50  [2.07; 17.06] | 15.72  [2.51; 34.50] | 9.65  [2.70; 29.25] | 7.45  [0.99; 35.02] | 0.689 | - | - | - | - | - | - |
| CD1c | 10.32  [1.52; 22.66] | 10.00  [1.89; 31.00] | 10.49  [1.43; 27.78] | 3.72  [0.92; 29.89] | 0.784 | - | - | - | - | - | - |
| CD25 | 4.00  [0.41; 20.16] | 14.75  [0.32; 35.46] | 5.01  [0.01; 28.59] | 10.45  [0.12; 35.13] | 0.793 | - | - | - | - | - | - |
| CD49e | 14.28  [7.00; 33.87] | 29.00  [12.75; 55.67] | 19.60  [9.67; 59.45] | 15.47  [9.66; 40.99] | 0.268 | - | - | - | - | - | - |
| ROR1 | 10.44  [1.97; 25.67] | 25.13  [7.47; 47.65] | 18.61  [2.61; 33.76] | 17.00  [0.64; 39.09] | 0.368 | - | - | - | - | - | - |
| CD209 | 7.67  [2.08; 27.53] | 11.25  [3.75; 47.17] | 6.50  [1.94; 31.56] | 8.32  [0.92; 36.60] | 0.804 | - | - | - | - | - | - |
| CD9 | 69.10  [51.99; 92.39] | 74.13  [56.64; 97.87] | 68.29  [55.33; 106.56] | 80.62  [58.07; 119.07] | 0.690 | - | - | - | - | - | - |
| SSEA-4 | 4.21  [0.39; 18.77] | 9.95  [0.31; 22.20] | 24.80  [5.76; 57.44] | 22.51  [9.12; 32.60] | **0.003** | 1.000 | **0.027** | **0.035** | 0.064 | 0.082 | 1.000 |
| HLA-ABC | 9.66  [2.53; 36.92] | 22.98  [11.71; 43.76] | 16.04  [6.00; 33.65] | 14.17  [5.18; 27.88] | 0.286 | - | - | - | - | - | - |
| CD63 | 111.74  [84.15; 161.25] | 116.05  [87.92; 158.25] | 124.84  [92.10; 164.69] | 136.43  [105.97; 156.67] | 0.747 | - | - | - | - | - | - |
| CD40 | 27.90  [12.99; 72.22] | 85.60  [62.37; 116.33] | 52.32  [20.80; 71.01] | 27.40  [16.81; 54.49] | **<0.001** | **<0.001** | 1.000 | 1.000 | **0.002** | **<0.001** | 1.000 |
| CD62P | 120.62  [69.34; 228.33] | 645.05  [265.65; 1275.97] | 253.15  [135.57; 511.99] | 327.44  [172.00; 499.56] | **<0.001** | **<0.001** | **0.020** | **0.005** | **0.009** | **0.032** | 1.000 |
| CD11c | 12.17  [6.23; 25.17] | 20.64  [4.68; 42.96] | 14.01  [1.73; 34.74] | 7.15  [1.83; 22.72] | 0.487 | - | - | - | - | - | - |
| CD81 | 117.68  [42.87; 137.76] | 89.63  [66.84; 122.10] | 86.16  [42.13; 125.25] | 71.71  [36.46; 99.44] | 0.314 | - | - | - | - | - | - |
| MCSP | 6.03  [0.59; 28.04] | 25.30  [7.00; 45.88] | 5.52  [0.58; 34.50] | 9.09  [0.19; 32.49] | 0.376 | - | - | - | - | - | - |
| CD146 | 4.41  [1.77; 10.95] | 6.35  [1.00; 18.33] | 1.12  [0.00; 6.87] | 1.92  [0.42; 7.92] | 0.065 | - | - | - | - | - | - |
| CD41b | 39.66  [21.54; 64.34] | 94.56  [60.35; 177.84] | 64.24  [42.62; 91.29] | 47.21  [31.80; 82.45] | **<0.001** | **<0.001** | 0.289 | 1.000 | **0.027** | **<0.001** | 1.000 |
| CD42a | 139.48  [112.62; 231.07] | 561.26  [339.57; 1205.32] | 321.75  [171.21; 517.34] | 272.89  [154.31; 504.12] | **<0.001** | **<0.001** | **0.015** | **0.040** | 0.057 | **0.022** | 1.000 |
| CD24 | 10.18  [2.71; 36.92] | 35.59  [10.93; 55.25] | 18.84  [2.35; 53.36] | 15.67  [2.60; 48.54] | 0.193 | - | - | - | - | - | - |
| CD86 | 7.63  [1.72; 21.73] | 16.50  [4.35; 35.68] | 8.66  [0.40; 21.63] | 8.00  [0.19; 23.14] | 0.430 | - | - | - | - | - | - |
| CD44 | 8.59  [2.50; 26.51] | 16.23  [2.56; 39.23] | 8.11  [1.56; 33.34] | 12.73  [2.31; 31.82] | 0.937 | - | - | - | - | - | - |
| CD326 | 3.05  [0.97; 13.99] | 13.49  [0.00; 32.47] | 9.51  [1.66; 30.76] | 6.74  [0.49; 35.09] | 0.841 | - | - | - | - | - | - |
| CD133/1 | 12.86  [3.41; 24.81] | 26.00  [4.26; 64.06] | 15.00  [4.46; 42.37] | 11.73  [2.01; 29.35] | 0.521 | - | - | - | - | - | - |
| CD29 | 59.45  [34.05; 156.13] | 80.91  [56.24; 118.37] | 82.36  [51.18; 117.97] | 82.25  [48.92; 157.11] | 0.663 | - | - | - | - | - | - |
| CD69 | 23.95  [11.38; 42.97] | 32.95  [21.16; 57.50] | 24.28  [10.06; 60.99] | 23.52  [9.88; 64.66] | 0.347 | - | - | - | - | - | - |
| CD142 | 7.65  [0.98; 34.73] | 20.50  [4.43; 41.03] | 8.20  [0.49; 51.43] | 16.44  [0.49; 45.80] | 0.580 | - | - | - | - | - | - |
| CD45 | 6.25  [0.48; 19.00] | 15.07  [2.44; 28.92] | 4.14  [1.10; 24.32] | 6.78  [1.65; 26.31] | 0.395 | - | - | - | - | - | - |
| CD31 | 38.67  [21.07; 68.79] | 86.72  [59.75; 131.05] | 60.78  [29.24; 98.40] | 78.54  [39.37; 103.90] | **0.001** | **<0.001** | 0.376 | 0.081 | 0.180 | 0.712 | 1.000 |
| CD20 | 5.96  [0.90; 26.01] | 17.63  [3.00; 43.44] | 14.99  [1.55; 49.73] | 11.36  [0.27; 40.02] | 0.419 | - | - | - | - | - | - |
| CD14 | 12.38  [3.94; 27.29] | 8.46  [3.61; 36.73] | 10.66  [0.75; 21.24] | 7.43  [0.89; 24.46] | 0.632 | - | - | - | - | - | - |

Data from Flow Cytometry (FC) analysis. Subjects with ST-segment elevation myocardial infarction (STEMI) were compared to controls (CTRL), from the training cohort. STEMI patients were evaluated before percutaneous coronary intervention (pre-PCI) and after 24 or 48 hrs from reperfusion. MFI (Median Fluorescence Intensity). Data are expressed as median and interquartile range. *P-*values of less than 0.05 were considered significant and indicated by bold characters.

***Extended Methods***

*Patient management and data extraction*

We recruited 98 subjects at the Fondazione Cardiocentro Ticino, Lugano (Switzerland): 30 controls (Ctrl), 30 patients with a diagnosis of ST-segment elevation myocardial infarction (STEMI), and 38 with chronic coronary artery disease and stable angina (SA) [1,2]. STEMI patients were evaluated before primary percutaneous coronary intervention (PCI) and 24 hrs and 48 hrs after reperfusion; hence, a total of 158 samples were analyzed. A second independent cohort was enrolled at the Città della Salute e della Scienza, University of Torino (Italy): 40 Ctrl and 40 patients with STEMI evaluated before PCI.

For STEMI patients, PCI performed immediately after hospital admission confirmed at least 1 culprit coronary lesion, treated with PTCA/stenting. All patients received standard dual anti-thrombotic therapy (aspirin 150-300 mg and one of the following drugs: clopidogrel 300-600 mg prasugrel 60 mg, or ticagrelor 180 mg), associated with unfractionated heparin 70-100 IU/Kg.

Ctrl were enrolled after exclusion of coronary stenosis (>30% lumen diameter reduction) by coronary angiography computer tomography. SA patients showed at least one significant coronary stenosis (>70% diameter reduction) by coronary angiography.

For each patient, we report age (years), sex, familiarity for CAD (yes/no), diagnosis of hypertension (yes/no), diabetes (yes/no), dyslipidemia (yes/no), chronic kidney disease (CKD; yes/no), smoking habit (yes/no), systolic and diastolic blood pressure (BP; mmHg), weight (Kg), BMI (Kg/m^2^), high-sensitive (hs) troponin (ng/L), white blood cells (WBC; n/L), creatinine (mg/dL), glomerular filtration rate (GFR; mL/min), C-reactive protein (CRP; mg/L), glycemia (mmol/L), total cholesterol (mmol/L), HDL (mmol/L), triglycerides (mmol/L), and ejection fraction (EF; %) at echocardiography (Table 1). In patients with STEMI, we also evaluated time after onset of chest pain (hrs), hs troponin 24 and 48 hrs after PCI, the infarcted myocardial region, and the vessel culprit lesion.

*Extracellular vesicles characterization*

Samples underwent serial differential centrifugations prior to analysis: 1,600 g for 15 min at 4°C to remove intact cells, and then 3,000 g for 20 min, 10,000 g for 15 min, and 20,000 g for 30 min to remove cellular debris, and larger EVs (Figure S1a).

Circulating serum extracellular vesicles (EV) were visualized and quantified through nanoparticle tracking analysis (NTA) using NanoSight LM10 (*Malvern Instruments, United Kingdom*) equipped with a 405 nm laser and Nanoparticle Tracking Analysis NTA 2.3 analytic software [3]; 1 µL of serum was diluted 1:1000 in 999 µL phosphate buffered saline (PBS) sterile solution and exposed to a laser light source. Brownian movements of EVs were recorded by a camera and size and number of EVs per mL were calculated by Stokes-Einstein equation. Three videos of 60 s were recorded for each sample to perform the analyses. EV size was measured using the whole 30-1000 nm scale; however, the number of EV >500 nm in size was negligible (<0.01% of all EVs), and therefore we used a smaller scale to show the data (NTA graphs shown in Figures 1 and S1b ranged between 30 and 500 nm).

All samples underwent bead-based EV-capture and analysis by flow cytometry (FC), using MACSPlex human Exosome Kit (*Miltenyi Biotec; Bergisch Gladbach, Germany*). Median fluorescence intensity (MFI) was measured by the MACSQuant Analyzer 10 flow cytometer (*Miltenyi Biotec; Bergisch Gladbach, Germany*), as previously described [4,5]. The multiplex platform is based on 4.8 um diameter polystyrene beads, labelled with different amounts of 2 dyes (phycoerythrin [PE] and fluorescein isothiocyanate [FITC]), to generate 37 different bead-subsets discriminable by FC analysis. Each bead subset is conjugated with a different capture antibody, that recognizes EVs carrying the respective antigen. Antigens included in the multiplex EV analysis are reported in Table S2. After beads+sample incubation, EVs bound to beads were detected by allophycocyanin [APC]-conjugated anti-CD9, anti-CD63, and anti-CD81 antibodies. MACSPlex Exosome Setup Beads were used to setup the MACSQuant analyzer: a trigger for the side scatter (SSC) and the forward scatter (FSC) was selected to confine the measurement on capture beads; single beads were gated to exclude doublets and non-bead events. FITC and PE voltage were adapted to optimize the discrimination of the 37 bead subsets; single bead subsets were finally each gated to allow the measurement of the APC median fluorescence signal intensity.

Samples were processed as follow (see also Figure S1): 50 uL of serum (containing a number of EVs per mL ranging between 1.0E+9 e 5.0E+10) were diluted to a final volume of 120 uL with MACSPlex buffer (MPB) and incubated overnight (14-16 h) protected from light on an orbital shaker (800 rpm at 10°C) with MACSPlex Exosome Capture Beads containing the 37 antibody-coated bead subsets. MPB was used as blank control. To wash beads, 1 mL of MPB was added to each tube and then centrifuged 3.000 g for 10 minutes at 10°C. After careful aspiration of 1 mL of supernatant, 15 uL of MACSPlex Exosome Detection Reagent (5 uL for each APC-conjugated anti-CD9, anti-CD63, and anti-CD8 antibody) were added and incubated for 1 hour protected from light on an orbital shaker (450 rpm at 10°C). After another washing step (see before), 1 mL of MPB was added to each tube and samples were incubated for 15 minutes protected from light on an orbital shaker (450 rpm at 10°C). Tubes were then centrifuged 3.000 g for 10 minutes at 10°C and 1 mL supernatant was aspirated, leaving 150 uL in the tube. Samples were manually mixed immediately before 100-120 uL were loaded to and acquired by the instrument, resulting in approximately 10.000-15.000 single bead events being recorded for each sample. APC-MFI was corrected by subtracting the respective fluorescence value of blank control and then normalized by the mean MFI of CD9, CD63, and CD81 subsets.

Using the above protocol, serum samples were directly processed without EV enrichment and purification steps by other methods. To rule out confounding effects due to methodological issues, we compared the standard procedure described above (beads immuno-capture) with EV isolation by ultracentrifugation and size-exclusion chromatography (SEC). Briefly, samples underwent ultracentrifugation at 100,000 g for 3 hours at 4-10 °C; pellet was resuspended in 100 uL PBS prior to further analyses. SEC was performed by qEV Size Exclusion Column (*iZON science, Oxford, UK*), according to manufacturer’s instructions (Figure S2a and S2b). Moreover, we compared serum and plasma samples (Figures S2c and S3) and different duration of the incubation with capture beads (overnight vs. 1-hour incubation; Figure S2D). Technical reproducibility of the flow cytometric assays was evaluated by analyzing duplicates of the same sample in different days (Figure S4).

*Statistical analysis*

IBM SPSS Statistics 22 (IBM Corp., Armonk, New York, USA) and GraphPad PRISM 7.0a (La Jolla, California, USA) were used for statistical analyses.

Scalar variables were analyzed with the Kolmogorov–Smirnov test to determine their distributions. Normally distributed variables were expressed as mean ± standard deviation and were analyzed by ANOVA and post-hoc Bonferroni’s tests; non-normally distributed variables were expressed as median [interquartile range] and were analyzed by Kruskal-Wallis’s test. Categorical variables were expressed as absolute number (percentage) and were compared through a chi square test (Fisher’s exact test when sample size was ≤ 5).

Correlations were evaluated by Pearson’s test (R coefficient) and regression curve analyses. Receiver operating characteristics (ROC) curve was used to assess the area under the curve (AUC) and to compare diagnostic performances of selected variables; the Younden Index (J = sensitivity + specificity - 1) was calculated to determine the cut-off with the higher accuracy. Multivariate logistic regression analysis was performed to determine odds ratio (ORs); an OR greater than 1 indicates an increased likelihood of STEMI diagnosis, an OR less than 1, a decreased likelihood. *P-*values of less than 0.05 were considered significant.

*Diagnostic modelling*

Supervised machine learning algorithms were used to build the diagnostic model; analyses were performed with Python 3.5 (library, scikit-learn). The algorithms formulate predictions about future instances basing on a given set of labeled paired input-output training sample data. Briefly, a linear discriminant analysis (LDA) was performed as feature reduction strategy to create a canonical plot (Figure 5a) representing patients stratified according to their diagnosis and the expression of EV-surface epitopes. LDA employs linear combinations of variables to maximize the separation between groups by increasing precision estimates with variance reduction [6]. The algorithm computes a set of coefficients for linear combination of each variable to estimate the diagnosis. The estimation is derived from the following equation: Diagnosis = LDA_coeff1_*Variable_1_ + LDA_coeff2_*Variable_2_ + … + LDA_coeffn_*Variable_n_ > selected cut-off. The canonical axes of the plot are calculated by the LDA from weighted linear combinations of variables included in the model. The crosses indicate the means of (canonical 1; canonical 2) for each group, the ellipses include patients with a linear combination coefficient that falls within the mean +/- SD (canonical 1 +/- SD; canonical 2 +/- SD); each patient is indicated by a point.

The diagnostic models were built through a random forest (RF) classification algorithm to discriminate patients with a diagnosis of STEMI from controls. The algorithm created 20 different classification trees with a maximum number of 12 splits for each tree. The predicted diagnosis was based on the outcome of each classification tree of the RF: for example, if at least 11 of 20 trees of the RF predict a diagnosis of STEMI, the patient will be classified as STEMI. We built 2 RF models using the full panel of EV epitopes, or 5 selected EV epitopes. The models were created on a training cohort (STEMI and Ctrl patients from the Fondazione Cardiocentro Ticino, Lugano – Switzerland; n=60) and tested on an independent validation cohort (STEMI and Ctrl patients from the Città della Salute e della Scienza, University of Torino – Italy; n=80). A representative classification tree for each RF model is reported in Figure 5c and 5e. An overfitting bias was defined as the difference between the accuracy of the RF model on the training cohort and the accuracy on the validation cohort.

**REFERENCES**

1. Ibanez B, James S, Agewall S, Antunes MJ, Bucciarelli-Ducci C, Bueno H, Caforio ALP, Crea F, Goudevenos JA, Halvorsen S, Hindricks G, Kastrati A, Lenzen MJ, Prescott E, Roffi M, Valgimigli M, Varenhorst C, Vranckx P, Widimský P; ESC Scientific Document Group. 2017 ESC Guidelines for the management of acute myocardial infarction in patients presenting with ST-segment elevation: The Task Force for the management of acute myocardial infarction in patients presenting with ST-segment elevation of the European Society of Cardiology (ESC). *Eur Heart J* 2018;39:119-177.
2. Montalescot G, Sechtem U, Achenbach S, Andreotti F, Arden C, Budaj A, Bugiardini R, Crea F, Cuisset T, Di Mario C, Ferreira JR, Gersh BJ, Gitt AK, Hulot JS, Marx N, Opie LH, Pfisterer M, Prescott E, Ruschitzka F, Sabaté M, Senior R, Taggart DP, van der Wall EE, Vrints CJ; ESC Committee for Practice Guidelines, Zamorano JL, Achenbach S, Baumgartner H, Bax JJ, Bueno H, Dean V, Deaton C, Erol C, Fagard R, Ferrari R, Hasdai D, Hoes AW, Kirchhof P, Knuuti J, Kolh P, Lancellotti P, Linhart A, Nihoyannopoulos P, Piepoli MF, Ponikowski P, Sirnes PA, Tamargo JL, Tendera M, Torbicki A, Wijns W, Windecker S; Document Reviewers, Knuuti J, Valgimigli M, Bueno H, Claeys MJ, Donner-Banzhoff N, Erol C, Frank H, Funck-Brentano C, Gaemperli O, Gonzalez-Juanatey JR, Hamilos M, Hasdai D, Husted S, James SK, Kervinen K, Kolh P, Kristensen SD, Lancellotti P, Maggioni AP, Piepoli MF, Pries AR, Romeo F, Rydén L, Simoons ML, Sirnes PA, Steg PG, Timmis A, Wijns W, Windecker S, Yildirir A, Zamorano JL. 2013 ESC guidelines on the management of stable coronary artery disease: the Task Force on the management of stable coronary artery disease of the European Society of Cardiology. *Eur Heart J* 2013;34:2949-3003.
3. Dragovic RA, Gardiner C, Brooks AS, Tannetta DS, Ferguson DJ, Hole P, Carr B, Redman CW, Harris AL, Dobson PJ, Harrison P, Sargent IL. Sizing and phenotyping of cellular vesicles using Nanoparticle Tracking Analysis. *Nanomedicine* 2011;7:780-788.
4. Koliha N, Wiencek Y, Heider U, Jüngst C, Kladt N, Krauthäuser S, Johnston IC, Bosio A, Schauss A, Wild S. A novel multiplex bead-based platform highlights the diversity of extracellular vesicles. *J Extracell Vesicles* 2016;5:29975.
5. Wiklander OPB, Bostancioglu RB, Welsh JA, Zickler AM, Murke F, Corso G, Felldin U, Hagey DW, Evertsson B, Liang XM, Gustafsson MO, Mohammad DK, Wiek C, Hanenberg H, Bremer M, Gupta D, Björnstedt M, Giebel B, Nordin JZ, Jones JC, El Andaloussi S, Görgens A. Systematic Methodological Evaluation of a Multiplex Bead-Based Flow Cytometry Assay for Detection of Extracellular Vesicle Surface Signatures. *Front Immunol* 2018;9:1326.
6. James G, Witten D, Hastie T, Tibshirani R. Chapter 4.4: Linear discriminant analysis. In: An Introduction to Statistical Learning. Springer texts in Statistics (reprinted). 2017; 138-150.
